# Inside the Microreactor: In Situ Real‐Time Observation of Vapor–Liquid–Solid Growth of Monolayer TMDCs

**DOI:** 10.1002/advs.202516784

**Published:** 2025-12-12

**Authors:** Hiroo Suzuki, Yutaro Senda, Kaoru Hisama, Shuhei Aso, Yuta Takahashi, Shun Fujii, Yasuhiko Hayashi

**Affiliations:** ^1^ Graduate School of Environment Life and Natural Science and Technology Okayama University Okayama 700‐8530 Japan; ^2^ Faculty of Environment Life and Natural Science and Technology Okayama University Okayama 700‐8530 Japan; ^3^ Department of Electrical and Communication Engineering School of Engineering Okayama University Okayama 700‐8530 Japan; ^4^ Research Initiative for Supra‐Materials Shinshu University Nagano 380‐8553 Japan; ^5^ Department of Physics Faculty of Science and Technology Keio University Yokohama 223‐8522 Japan; ^6^ Present address: Preferred Networks, Inc. 1‐6‐1, Otemachi, Chiyoda‐ku Tokyo 100‐0004 Japan

**Keywords:** in situ observation, microreactor, nanoribbon, transition metal dichalcogenides, vapor–liquid–solid growth

## Abstract

Real‐time observation of molten droplet–driven crystal growth provides an unprecedented in situ window into the formation of atomically thin transition metal dichalcogenides (TMDCs). Materials such as MoS_2_ and WS_2_ exhibit remarkable optoelectronic properties arising from their monolayer structures, enabling advanced applications that exploit valley degrees of freedom. Among various synthetic approaches, vapor–liquid–solid (VLS) growth from a low‐melting molten source containing alkali, transition metal, halide, and oxygen atoms has proven highly effective for producing large single‐crystal monolayer TMDCs, while also yielding distinct growth regimes including molten particle–driven nanoribbon formation. A chemical vapor deposition method is recently developed that integrates VLS growth with the spatial confinement provided by a substrate‐stacked microreactor; however, the precise role of confinement and droplet dynamics remains unclear. Here, in situ the VLS growth of TMDCs inside such microreactors is directly captured using an infrared heating furnace. The microreactor, formed by sealing a transparent sapphire substrate with a Na_2_WO_4_‐coated SiO_2_/Si wafer, enables continuous observation of growth mode transitions governed by the balance of sulfur and Na_2_WO_4_ supply. The findings demonstrate that fine control over precursor supply rates is essential for engineering the size, morphology, and crystallinity of monolayer TMDCs in the VLS regime.

## Graphical abstract

In situ observation of VLS growth in a confined microreactor reveals various growth modes of monolayer TMDCs, including abnormal ribbon growth and particle‐driven growth. The confined space and precursor balance influence droplet behavior and growth dynamics, offering new insights into the controlled synthesis of TMDCs via the VLS mechanism.

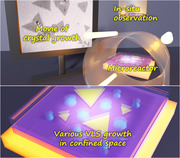

## 1 Introduction

Atomically thin transition metal dichalcogenides (TMDCs), such as MoS_2_ and WS_2_, have emerged as promising semiconducting materials due to their unique layer‐dependent electronic structures. Notably, these materials undergo a transition from an indirect to a direct bandgap when reduced from a multilayer to a monolayer form, leading to enhanced light–matter interactions.^[^ ^1^, ^2^ ^]^ As a result, monolayer TMDCs exhibit remarkable optoelectronic characteristics, including pronounced photoluminescence (PL), electroluminescence, and high absorption coefficients.^[^ ^3^, ^4^, ^5^ ^]^ Their atomic‐scale thickness and exceptional mechanical flexibility make TMDCs ideal candidates for next‐generation flexible optoelectronic devices, such as photodetectors, light‐emitting diodes, and solar cells.^[^ ^5^, ^6^, ^7^, ^8^, ^9^ ^]^ In addition, TMDCs are gaining attention as a beyond‐silicon channel material for field‐effect transistors possessing an ultimate thin structure of less than 1 nm in cutting‐edge integrated circuits.^[^ ^10^, ^11^, ^12^, ^13^ ^]^ Furthermore, TMDCs provide a versatile platform for exploring novel physical phenomena, particularly in the field of valleytronics, which exploits the valley degree of freedom associated with the inequivalent K and K′ points in momentum space.^[^ ^14^ ^]^ The ability to manipulate valley polarization opens avenues for advanced quantum information technologies through the coupling between valley states and photons. Owing to these excellent properties, monolayer TMDCs are an essential building block of 2.5D materials,^[^ ^15^, ^16^ ^]^ represented by the vertically stacked constrictions of 2D materials,^[^ ^17^, ^18^ ^]^ especially with a twist angle degree of freedom.^[^ ^19^, ^20^, ^21^ ^]^ Chemical vapor deposition (CVD) has been widely employed for the large‐scale synthesis of monolayer TMDC films and single crystals, serving as a key technique for realizing their integration into practical devices. In early studies on TMDC growth via CVD, powdered metal oxides such as WO_3_ and MoO_3_ were commonly used as transition metal precursors for WS_2_ and MoS_2_, respectively.^[^ ^22^, ^23^ ^]^ However, their inherently low chemical reactivity—stemming from their high melting and boiling points—has posed challenges to achieving reproducible and consistent TMDC growth.

Salt‐assisted growth and vapor–liquid–solid (VLS) growth have been reported as effective strategies for the growth of TMDC crystals using low‐melting materials composed of alkali, transition metal, halide, and oxygen atoms.^[^ ^24^, ^25^, ^26^, ^27^, ^28^, ^29^ ^]^ In salt‐assisted growth, mixtures of transition metal oxides (for example, MoO_3_ and WO_3_) and salts (for example, NaCl, KCl, and NaF)^[^ ^24^, ^25^, ^26^, ^27^, ^28^, ^29^ ^]^ are used. The salts promote the formation of volatile intermediates or eutectic liquids, thereby enhancing mass transport and crystal nucleation. In VLS growth, pre‐formed alkali transition metal oxides (for example, Na_2_MoO_4_ and Na_2_WO_4_)^[^ ^30^, ^31^ ^]^ serve as molten precursors at the growth temperature. These molten droplets act as localized liquid media that mediate crystal nucleation and lateral growth at the liquid–solid interface. The VLS mechanism has enabled the growth of large‐area, high‐quality monolayer TMDC single crystals with high reproducibility.^[^ ^30^, ^31^ ^]^ Furthermore, VLS growth can achieve molten particle motion‐driven nanoribbon growth.^[^ ^27^ ^]^ The centimeter‐scale growth of monolayer single crystals of TMDC has been reported using 2D Czochralski growth based on VLS growth.^[^ ^32^ ^]^ However, the detailed mechanism of the VLS growth of monolayer TMDCs is not well understood. We have proposed a CVD method that combines VLS growth and the confined space of microreactors.^[^ ^33^ ^]^ A microreactor can provide a well‐controlled environment for the growth of 2D materials.^[^ ^34^, ^35^, ^36^, ^37^, ^38^, ^39^, ^40^, ^41^, ^42^, ^43^, ^44^ ^]^ Using the combined method, we grew millimeter‐scale monolayer TMDC single crystals.^[^ ^33^ ^]^ The growth mechanism of monolayer TMDC in the microreactor was investigated, and it was determined that surface diffusion‐limited growth governs the growth regime of monolayer TMDC. Although high‐quality monolayer TMDC single crystals can be obtained using this method, the growth process of TMDC crystals, including the presence of dynamic molten droplets, the effect of confined space, and the growth morphology during crystal growth, has not been well clarified. These factors interact with each other and result in highly complex crystal‐growth processes.

In situ observation is an extremely powerful tool for investigating the growth mechanism of nanomaterials such as nanowires,^[^ ^45^ ^]^ carbon nanotubes,^[^ ^46^ ^]^ and 2D materials,^[^ ^47^, ^48^, ^49^, ^50^, ^51^, ^52^ ^]^ including TMDCs.^[^ ^48^, ^49^, ^50^, ^51^, ^52^ ^]^ Despite its usefulness for investigating growth mechanisms, there are only a limited number of reports on the in situ observation of TMDC growth. In situ observation has revealed nucleation dynamics,^[^ ^48^, ^49^ ^]^ different growth modes corresponding to rate‐limiting steps,^[^ ^50^ ^]^ and liquid‐phase edge epitaxy.^[^ ^51^ ^]^ These reports have strongly contributed to the understanding of growth mechanisms and the control of growth properties. In this study, we addressed the entire system of VLS growth in a microreactor using an in situ observation technique and uncovered the various growth regimes of monolayer TMDC corresponding to the growth conditions, such as the amount of supplied raw materials.

## 2 Results and Discussion

### 2.1 Setup for In Situ Observation of VLS Growth in Microreactors

**Figure 1:**
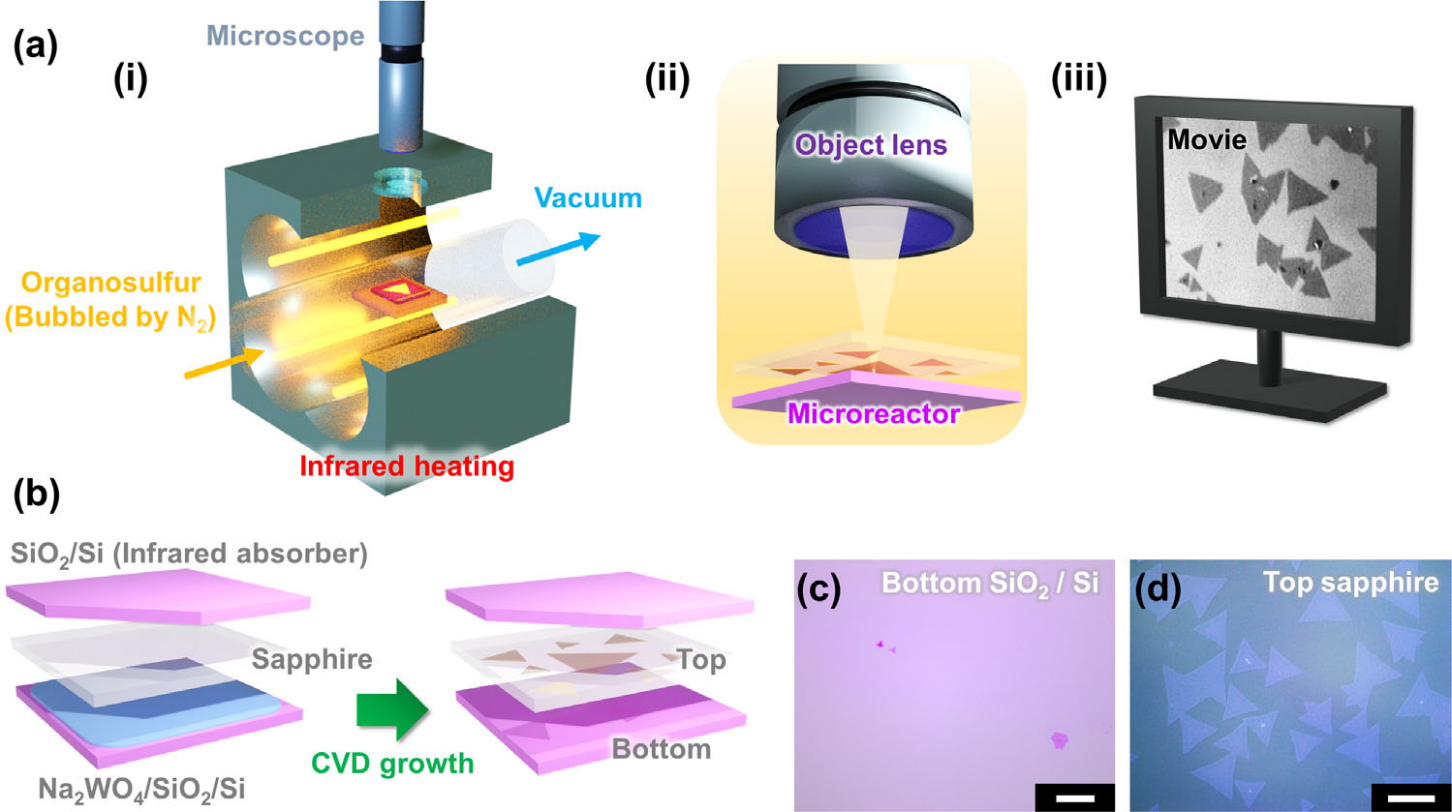
a) Schematic of the in situ observation CVD system. i) Infrared heating furnace equipped with an optical window for observing the inside of the chamber using an optical microscope. ii) Extracted illustration of microreactor constructed by Si (bottom) and transparent sapphire (top) substrate observed via the object lens of the optical microscope. iii) Real‐time monitoring of the crystal growth of monolayer TMDC. b) Configuration of microreactor constructed by (in order from the bottom to the top) a Na_2_WO_4_ solution spin‐coated onto a SiO_2_/Si substrate, a transparent sapphire substrate, and SiO_2_/Si substrate used to absorb infrared light, which was incomplete in one corner to enable in situ observation. After CVD growth, the monolayer WS_2_ crystals were dominantly grown on the top sapphire substrate. Micrographs of (c) bottom SiO_2_/Si substrate and (c) top sapphire substrate.

We constructed an in situ observation CVD system by combining a cold‐wall CVD setup and an optical microscope (**Figure** **1**a). The cold‐wall CVD setup was realized using an infrared heating furnace similar to that used in our previous report.^[^ ^33^ ^]^ Organosulfur (di‐tert‐butyl disulfide, DTBDS) was employed as a source of S and supplied via a bubbling technique using a N_2_ flow. The use of organosulfur as a S source can increase the reproducibility of TMDC growth compared with the use of evaporated S powder. The quartz chamber was vacuumed during the CVD process. Owing to the infrared heating system, which only uses heat infrared absorbers, such as carbon susceptors and Si substrates, the heat fluctuation of the gas flow, which prevents stable in situ observation, could be suppressed compared with the hot‐wall CVD system previously used in research on the in situ observation of TMDC growth.^[^ ^48^, ^49^, ^50^, ^51^ ^]^ This cold‐wall CVD system enables optical microscopy with less lens heating. The use of a vacuum system can also contribute to suppressing the heat fluctuation of the gas flow because of the lower gas flow rate compared with atmospheric CVD, which has also been mainly employed in previous research on the in situ observation of TMDC growth. The CVD system was equipped with a zoomable optical microscope with a large working distance (101 mm) to observe the growth substrate inside the CVD chamber through the optical window of the CVD furnace. To observe the interior of the microreactor, a transparent sapphire substrate was employed as the top substrate, covering the bottom SiO_2_/Si substrate (Figure 1b). We utilized aqueous Na_2_WO_4_ as the source of W for WS_2_ growth. The aqueous Na_2_WO_4_ solution with various concentrations (*C*_W_) was spin‐coated onto the bottom SiO_2_/Si substrate. We measured the morphology of Na_2_WO_4_ films spin‐coated onto SiO_2_/Si substrates with various coating solution concentrations (*C*_W_ = 1–5 mg mL^−1^) at both the center and edge regions, as shown in Figure S1 (Supporting Information). The film volume increased with increasing *C*_W_. Particulate forms were observed at *C*_W_ = 1 mg mL^−1^ (Figure S1a,b, Supporting Information), while island‐like structures were confirmed at *C*_W_ = 3 mg/mL (Figure S1c,d, Supporting Information). Film‐like structures appeared at *C*_W_ = 5 mg mL^−1^ (Figure S1e,f, Supporting Information), and their thicknesses were ≈3 and 15 nm at the center and edge regions, respectively. We constructed a sapphire/Na_2_WO_4_/(SiO_2_/Si) microreactor by covering the sapphire substrate with a Na_2_WO_4_ spin‐coated SiO_2_/Si substrate. Although the top sapphire substrate enabled in situ observation, it is not suitable for growth with an infrared heating system because it cannot absorb infrared light, preventing efficient heating in the infrared heating environment. Therefore, another SiO_2_/Si substrate was placed on top of the sapphire/Na_2_WO_4_/(SiO_2_/Si) microreactor as an infrared light absorber to efficiently heat the microreactor. We conducted in situ observation through part of this SiO_2_/Si substrate, as illustrated in Figure 1b. Figure 1c,d show micrographs of the surfaces of the bottom SiO_2_/Si (Figure 1c) and top sapphire substrates (Figure 1d) after the growth. Monolayer WS_2_ single crystals were predominantly grown on the top sapphire substrate (Figure 1c). However, few WS_2_ crystals grew on the bottom SiO_2_/Si substrate. This was due to the temperature difference between the two substrates. As mentioned above, the sapphire substrate has low absorption of infrared light (≈15%); therefore, the temperature of the top sapphire substrate was slightly lower than that of the bottom SiO_2_/Si substrate. Notably, we did not observe enhanced growth on the higher‐temperature side; instead, WS_2_ crystals preferentially grew on the lower‐temperature side (see Note S1, Supporting Information). When both the top and bottom sides were SiO_2_/Si substrates, monolayer WS_2_ single crystals grew on both the top and bottom SiO_2_/Si substrates (Figure S1, Supporting Information). It is worth noting that it is not always the case that WS_2_ crystals on the Na_2_WO_4_ spin‐coated bottom SiO_2_/Si substrate are larger than those on the top SiO_2_/Si substrate. The preferred side for the growth of larger crystals is sensitive to slight variations in the temperature difference between the top and bottom substrates. In a subsequent experiment, we conducted in situ observation of WS_2_ crystal growth on the surface of the top sapphire substrate.

### 2.2 Classification of Various Growth Regimes Corresponding to Growth Conditions

**Figure 2:**
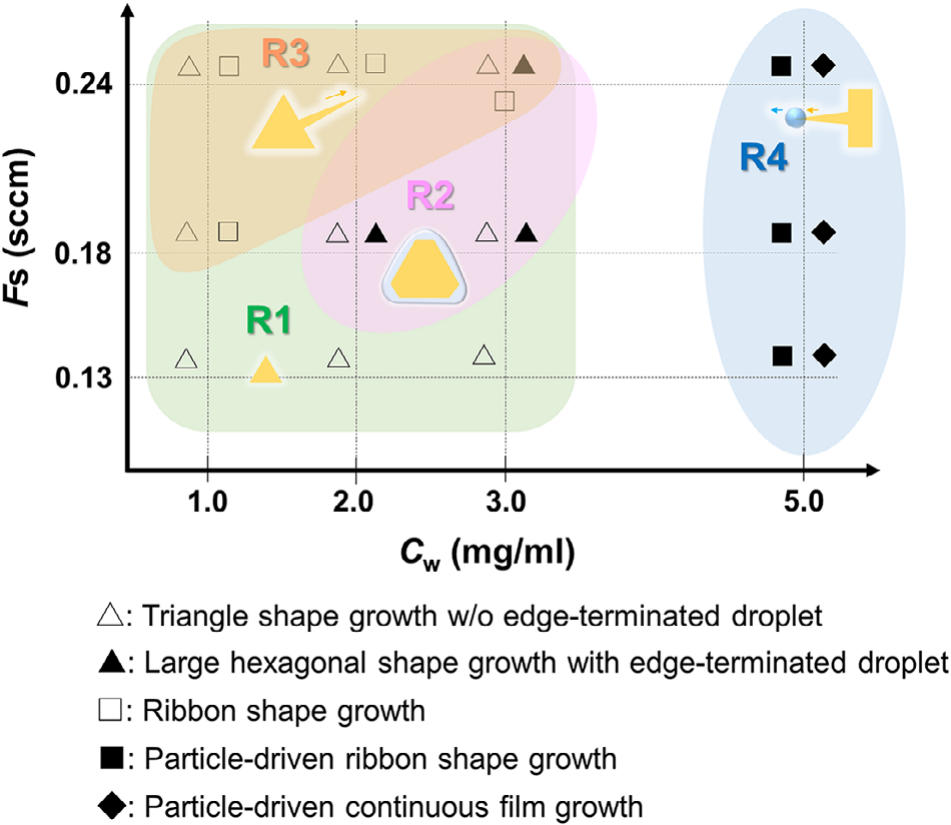
Area map of various growth regimes corresponding to different *C*_W_ and *F*_S_ conditions.

**Figure** **2** shows an area map classifying the various growth regimes corresponding to the *C*_W_ and the organosulfur flow rate (*F*_S_). We changed the *C*_W_ and *F*_S_ values from 1.0 to 5.0 mg mL^−1^ and from 0.13 to 0.24 sccm, respectively. In the wide‐range region where *C*_W_ was less than 3 mg mL^−1^, we observed the growth of triangular crystals, which is a typical growth regime of monolayer WS_2_ (regime 1, R1). In the relatively high‐*C*_W_ and ‐*F*_S_ region, we observed the growth of large triangular and hexagonal crystals (regime 2, R2). We observed the absence and presence of molten droplets at the edges during growth in R1 and R2, respectively. Under S‐rich conditions, abnormal growth such as ribbon‐shaped crystal growth (regime 3, R3) was observed. In the W‐rich region, molten particle‐driven growth of ribbon‐shaped crystals and a continuous film (regime 4, R4) were observed. As shown in Figure 2, the various VLS growth regimes can be controlled using the *C*_W_ and *F*_S_ conditions. We discuss the details of each growth regime in the following sections.

### 2.3 Comparison of Growth in R1 and R2

**Figure 3:**
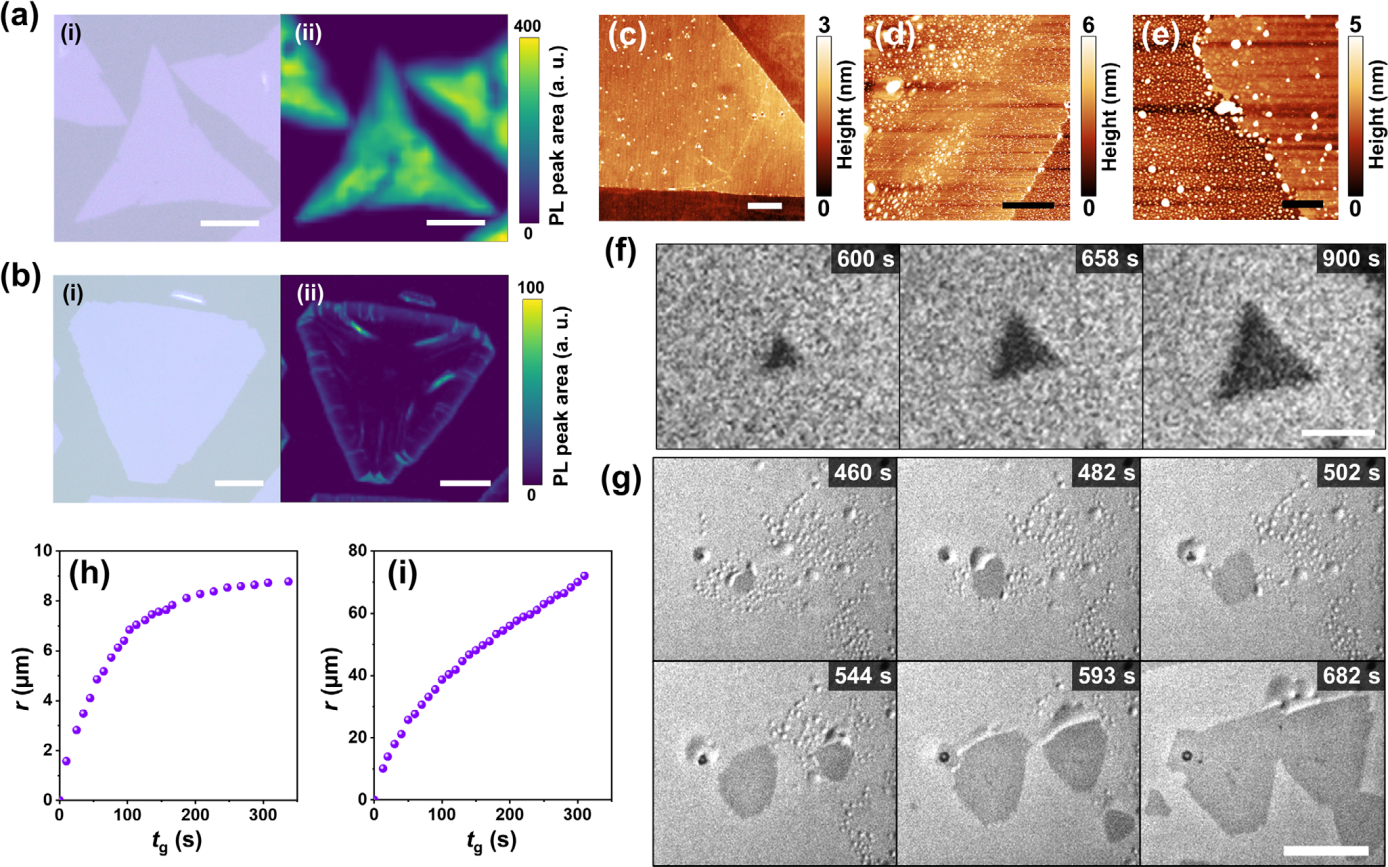
a,b) (i) Typical micrographs and (ii) their corresponding PL maps of monolayer WS_2_ crystals obtained in (a) R1 and (b) R2. Height images of monolayer WS_2_ grown in c) R1 and d,e) R2 taken with (d) low and e) high magnification. f,g) Time‐series micrographs of WS_2_ growth in (c) R1 and (d) R2, respectively. Process times are shown in each micrograph. *t*_g_ dependence of *r* in h) R1 and i) R2. The scale bars in (a–g) represent 10, 20 µm, 500 nm, 5 µm, 400 nm, 20 µm, and 100, respectively.

Typical crystals obtained in R1 and R2 are shown in **Figure** **3**a(i),b(i), respectively. Conventional triangular crystals of monolayer WS_2_ were obtained in R1 (Figure 3a(i)), and hexagonal crystals of monolayer WS_2_ were obtained in R2 (Figure 3b(i)). In R1, in situ observation revealed that the crystal maintained its triangular shape during growth (Figure 3f; Movie S1, Supporting Information). The process time elapsed in the time‐series micrographs are the time elapsed since the introduction of the organosulfur. In situ observation showed the growth of hexagonal‐like crystals in R2 (Figure 3g; Movie S2, Supporting Information). The crystal initially exhibited a round‐like shape at the early nucleation stage (465–502 s) and later transformed into a hexagon‐like shape (544–682 s). Nucleation occurred within droplets in a region abundant with them, and as the crystal grew, its edges pushed the droplets outward. To investigate the growth properties, we recorded the radius of the incircle of the crystal (*r*) at each growth time (*t*_g_) using the time‐series data shown in Figure 3f,g, where *t*_g_ is the time elapsed from nucleation. In the R1 (Figure 3h), the change in *r* gradually slowed as the growth progressed and eventually saturated. In R2 (Figure 3i), *r* increased quasi‐linearly within the observable time range up to *t*_g_ = 310 s. Owing to the presence of a droplet at the crystal edge during growth, the amount of source material supplied to the crystal to construct the crystal lattice remained almost constant as long as the droplet maintained its volume. The absence of a depression in the growth rate within the process time increased the crystal size obtained in R2.

Photoluminescence (PL) measurements were used to compare the quality of the WS_2_ crystals grown in R1 and R2 (Figure 3a(ii),b(ii); Figure S5, Supporting Information). The PL mappings indicate that the WS_2_ crystal grown in R1 has a higher and more uniform PL intensity than that grown in R2. Atomic force microscopy (AFM) was used to measure the surface topography of the crystals (Figure 3c–e). The AFM image of the crystal grown in R1 shows a relatively clean surface with a straight edge, although small particles were observed on the surface (Figure 3c). The thickness of the crystal is ≈0.65 nm, indicating a monolayer structure (Figure S6, Supporting Information). In contrast, the crystal grown in R2 was heavily contaminated with abundant residual precursors remaining on its surface (Figure 3d). The precursor particles were attached to the edges of the crystal (Figure 3e). These precursor particles may have contributed to the formation of irregularly shaped edges. From the in situ observation, we confirmed that the crystal grew from the nucleation site (Figure 3g), indicating single‐crystal growth rather than polycrystalline growth. Possible reasons for the extremely low PL intensity of the crystals grown in R2 are the higher growth velocity under R2 conditions, which introduces lattice defects, and the residual source materials, including Na, which induce strong doping of WS_2_.^[^ ^53^ ^]^ Both these effects led to a decrease in the PL intensity. The inclusion of Na‐rich residues in the WS_2_ domain can be explained by the overgrowth of WS_2_ on the Na─W─O─S molten phase, which can occur when the crystal growth rate is high, as has been previously reported.^[^ ^54^ ^]^ We confirmed this by the AFM phase imaging of residue‐rich crystals grown under the R2 condition (Figure S7, Supporting Information). Multiple particles with different contrasts in the AFM phase were observed within the domain. The small particles around the edges appear to be on top of the WS_2_ surface, as they show similar contrast to the particles on the sapphire substrate. In contrast, the particles in the interior region show no significant difference in contrast compared to the WS_2_ domain, indicating that these particles are covered by WS_2_ crystals. The confined Na─W─O─S molten phase could lead to strong doping of the WS_2_ crystals, resulting in a suppression of the PL intensity.

**Figure 4:**
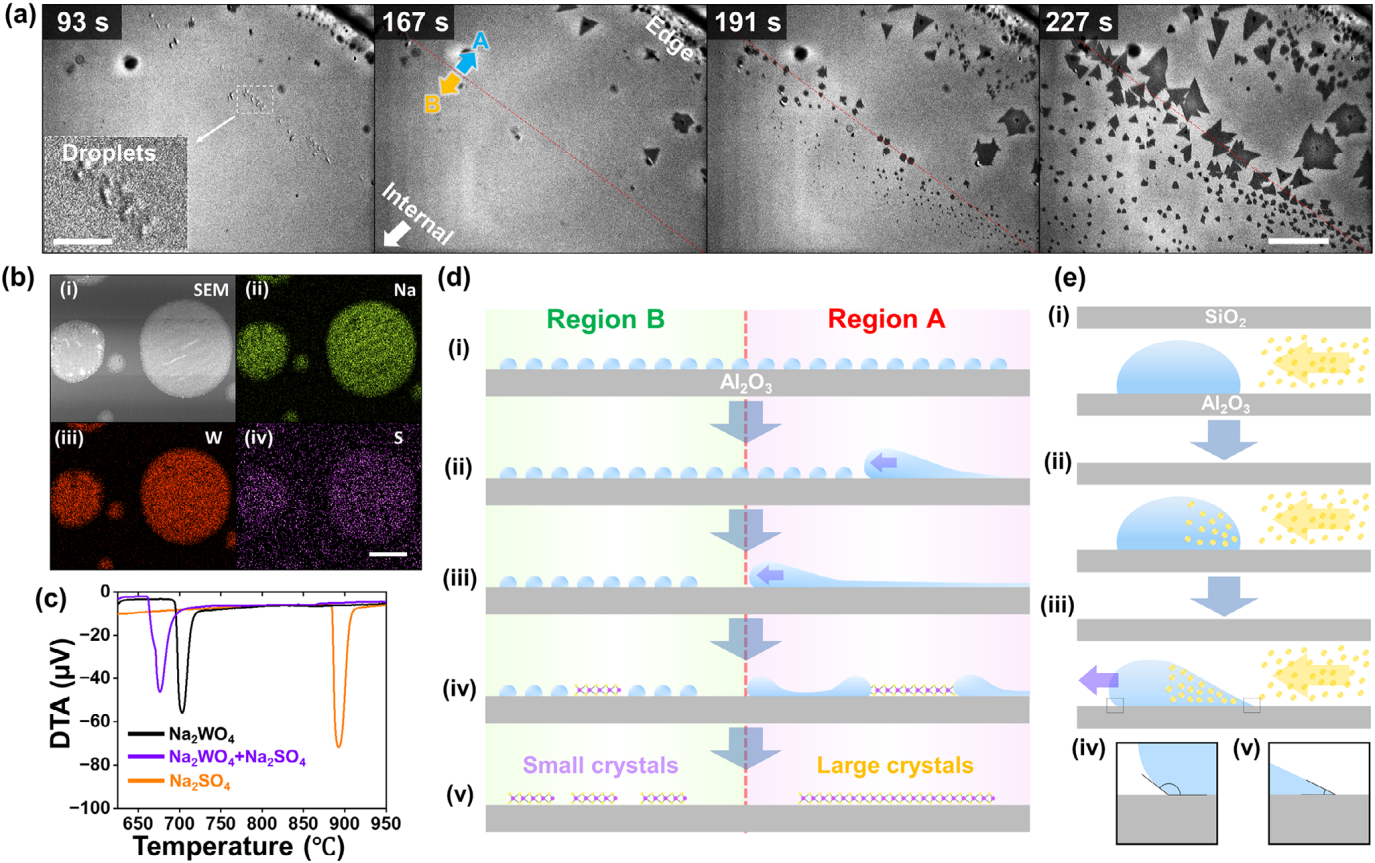
a) Time‐series micrographs near the edge of the microreactor, showing different A and B regions, corresponding to the areas where large and small crystals grow, respectively. The red line represents the droplet‐reach boundary. The inset shows a magnified image of the molten particles. Process times are shown in each micrograph. b) i) SEM image and SEM‐EDS mappings of ii) Na, iii) W, and iv) S. c) DTA spectra of Na_2_WO_4_, Na_2_WO_4_ + Na_2_SO_4_, and Na_2_SO_4_. d) Schematic of the growth model illustrating the different growth processes in regions A and B indicated in (a). e) Schematic of the model of the Marangoni effect‐driven molten droplet migration. The scale bars shown in (a), inset in (a), and (b) represent 200, 50, and 5 µm, respectively.

Next, we investigated the formation mechanism and dynamics of molten droplets in the microreactor, as well as their influence on crystal growth. After the introduction of the organosulfur, molten droplets initially formed near the edge of the microreactor (**Figure** **4**a; Movie S3, Supporting Information). Over time, these droplets migrated toward the interior of the microreactor. Notably, we did not observe droplet migration without the microreactor, indicating that the anisotropic organosulfur supply within the microreactor results in a sulfur concentration gradient that induces anisotropic droplet motion (see Note S2, Supporting Information). At a certain point, their movement ceased at a specific location, creating a droplet‐reach boundary (marked by the red line in Figure 4a) that divided the area into two distinct regions where the droplets have (Region A) and have not (Region B) passed, as shown in Figure 4a. Once the droplets reached the interior region, crystal growth commenced. A significant difference in growth behavior was observed between Regions A and B, resulting in relatively large (>100 µm in diameter) and small (<100 µm) crystals, respectively. These findings indicate that the presence of molten droplets facilitates the growth rate and size of WS_2_ crystals.

To investigate the elemental composition and thermodynamic properties of the molten liquid, we performed scanning electron microscopy combined with energy‐dispersive X‐ray spectroscopy (SEM‐EDS). The molten droplets were fabricated by introducing the organosulfur into the microreactor in the same manner as in the CVD process; however, the process was stopped before the onset of crystal growth. As mentioned above, molten droplets were formed upon the introduction of the organosulfur and migrated into the interior of the microreactor. Following this procedure, we obtained a solidified molten droplet after cooling to room temperature, as shown in Figure 4b(i). EDS mapping of Na, W, and S was performed (ignoring the O atoms from the sapphire substrate). A small amount of S (0.1 atom%, Figure S9, Supporting Information) was detected in addition to Na and W (Figure 4b). This small amount of S can be explained by the decrease in the S concentration in the droplet during the cooling process because the solubility of S atoms in the Na─W─O molten droplet decreased with decreasing growth temperature, leading to the underestimation of the S content in the Na─W─O─S molten droplet.

To investigate the influence of S in the molten droplet on its thermodynamic properties, we conducted differential thermal analysis (DTA) using Na_2_WO_4_, Na_2_SO_4_, and a Na_2_WO_4_ + Na_2_SO_4_ mixtures, which simulated an S‐Na‐W‐O molten phase at high temperatures (Figure 4c). The peak corresponding to the melting temperature of Na_2_WO_4_ was observed at 695.0 °C (Figure 4c). Despite Na_2_SO_4_ having a higher melting temperature (884.7 °C), this peak shifted to a lower temperature (661.5 °C) in the Na_2_WO_4_ + Na_2_SO_4_ mixture (the atomic ratio of W and S was adjusted to be 1:1). These results suggest that the Na─W─O─S molten phase formed by the incorporation of S into the Na‐W‐O molten phase has a lower melting temperature than that of the Na─W─O molten phase.

We considered a possible mechanism for the growth of the large crystals, as illustrated in Figure 4d,e. The Na_2_WO_4_ source can be transferred from the bottom SiO_2_ side to the top sapphire side by heating the microreactor (Figure S4, Supporting Information). Therefore, the motion of the molten droplets and the subsequent WS_2_ growth can primarily be considered to occur on the sapphire surface. To gain insight into the thermodynamics of molten droplets on SiO_2_/Si and sapphire, we analyzed the contact angle of Na_2_WO_4_ particles formed by annealing using cross‐sectional SEM, as shown in Figure S10 (Supporting Information). The contact angle of Na_2_WO_4_ on the SiO_2_ surface (10°) is significantly lower than that on the sapphire surface (60°). These trends were also confirmed by AFM, as shown in Figure S11 (Supporting Information). This indicates that Na_2_WO_4_ can spread and form a thin‐film structure on the SiO_2_ surface, whereas it tends to accumulate and form particle‐like structures on the sapphire surface. These differences in thermodynamic properties could contribute to the transfer of Na_2_WO_4_ from the bottom SiO_2_ side to the top sapphire side. The formation of thin Na_2_WO_4_ droplets on SiO_2_ could enhance evaporation due to the increased surface area and the nanoscale effect in the thin structure (measured diameter: ≈20 nm, Figure S11b,c, Supporting Information), which effectively reduces the melting point. In the initial stage, the Na_2_WO_4_ source was uniformly dispersed on the top sapphire substrate due to the transfer of Na_2_WO_4_ from the bottom SiO_2_/Si substrate. (Figure 4d(i)). When the organosulfur source was introduced into the microreactor, S atoms intercalated into the microreactor through the limited space between the top and bottom substrates (Figure 4e(i)). Consequently, S atoms were incorporated into the liquid‐state Na_2_WO_4_ particles (Figure 4e(ii)). This S incorporation led to the formation of Na─W─O─S molten droplets, and the transition from Na─W─O to Na─W─O─S resulted in a decrease in the melting point. This could be accompanied by an enhancement in fluidity and changes in the surface tension. Generally, high (low) surface tension in a droplet results in a symmetric spherical (asymmetric flattened) morphology. A moving asymmetric flattened droplet with an uneven contact angle was indeed observed, as shown in Figure S12 (Supporting Information). Furthermore, the introduction of organosulfur enhanced the wettability of molten Na_2_WO_4_ on the sapphire surface, whereas molten Na_2_WO_4_ alone exhibits low wettability on sapphire, as shown in Figure S10c,d (Supporting Information). The Na─W─O─S molten droplets formed during WS_2_ growth under sulfur‐rich conditions (*F*_S_ = 0.24 sccm, *C*_W_ = 1.0 mg/mL) exhibit flattened structures (Figure S13, Supporting Information), indicating high wettability to the sapphire substrate. These observations indicate that the incorporation of sulfur into Na_2_WO_4_ dramatically alters its thermodynamic properties, including its surface energy and interfacial energy with the substrate. We also experimentally observed a morphological change from asymmetric flattened to symmetric spherical, with high and low wettability, respectively, during the cooling process with a decrease in the atomic percentage of S in the molten droplet, as discussed above (Figure S14 and Movie S4, Supporting Information). This provides evidence that the introduction of S into the Na‐W‐O molten droplet decreased its surface tension. The DTA results suggest that both Na─W─O and Na─W─O─S could be in the molten state. However, significant differences exist in the thermodynamic behavior of the Na─W─O and Na─W─O─S molten states. In fact, the introduction of organosulfur into Na─W─O molten enhanced its fluidity, leading to the formation of a large molten droplet, which is clearly observed in in situ observation (Figure S8a, Supporting Information). In the microreactor, the organosulfur is supplied from the gap of the microreactor and penetrates toward the inward of the microreactor. Therefore, in the initial stage of the growth process, the molten droplets form near the edge region of the microreactor.

The mechanism of the migration of molten droplets into the microreactor interior was further investigated. Because S was supplied from outside the microreactor, an S concentration gradient was formed within the molten droplets. Assuming that an increase in the S concentration decreased the surface tension of the molten droplet, this causes asymmetry in both the surface tension and contact angle of the molten droplet, resulting in an asymmetric droplet morphology (Figure 4e(iii)) and an uneven contact angle (Figure 4e(iv,v)). The asymmetric surface tension within the molten droplets ultimately drives the droplet migration via the Marangoni effect.^[^ ^55^ ^]^ Briefly, the S concentration gradient, which induces an asymmetric surface tension, drives the migration of the molten droplets into the interior of the microreactor (Figure 4d(ii)). During droplet migration, owing to the high wettability of Na─W─O─S molten droplets, a thin‐film structure may form, as illustrated in Figure 4d(iii). In the crystal growth phase, WS_2_ crystals nucleate within the thin molten film and grow in the presence of abundant Na─W─O─S (Figure 4d(iv,v)). This results in growth under R2 conditions, which enhances the crystal size and suppresses the nucleation density. It has been reported that the formation of a thin‐film structure enhanced the crystal size during 2D Czochralski growth.^[^ ^32^ ^]^ In contrast, in region B, molten Na─W─O─S appears to have lower fluidity, which may result in the growth of small crystals and high nucleation density, similar to growth in R1 (Figure 4d(iv,v)). We further considered the possibility of droplet migration by the gas flow. Figure S15 (Supporting Information) shows the top‐view layout of the in situ observation. The directions of gas flow and droplet migration are either orthogonal or antiparallel. This indicates that the influence of gas flow on droplet migration is not significant in our experiments.

**Figure 5:**
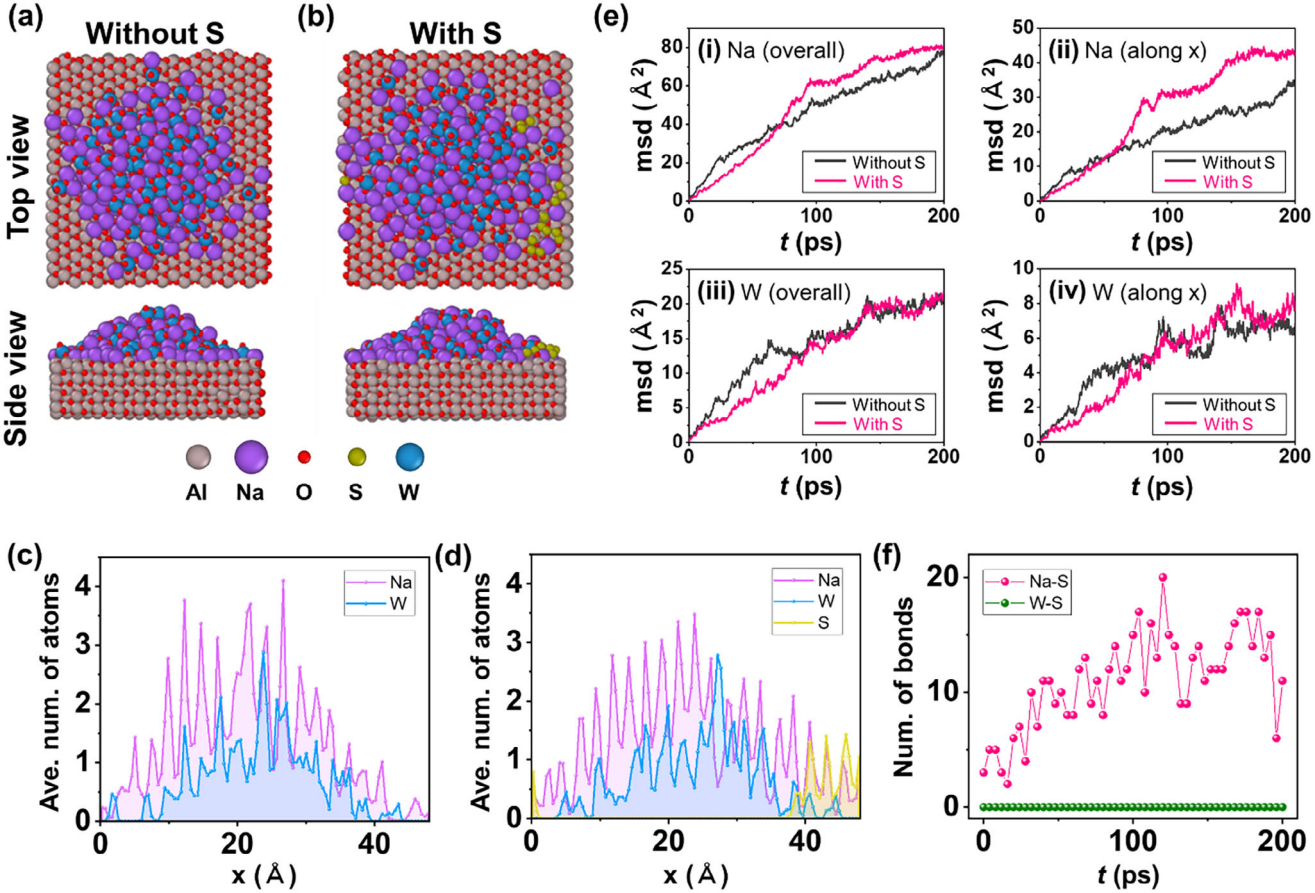
Top (*xy* plane) and front (*xz* plane) view of the snapshot of the MD simulation after 0.2 ns a) without and b) with two S8 rings adsorbed (See the legend for the colors of each element.), and the corresponding histograms of the distribution of atoms along the *x* axis during the last 0.1 ns simulation is shown in c, d), respectively. e) MSD as a function of time during the MD simulation. Panels i), ii), iii), and iv) show the Na (overall), Na (along the *x*‐axis), W (overall), and W (along the *x*‐axis), respectively. (f) Number of S─Na and S─W bonds shorter than 3.0 Å sampled every 4 ps as a function of time. The snapshots in (a) and (b) were visualized using the OVITO package.^[^ ^56^ ^]^

Molecular dynamics (MD) simulations were performed to obtain atomic‐scale insights into the thermodynamic and chemical behavior of Na‐W‐O‐S molten droplets during the initial stage of the dissociation of Na_2_WO_4_ in the CVD process (**Figure** **5**). As previously mentioned, the Na_2_WO_4_ source can be transferred from the bottom SiO_2_ side to the top sapphire side by heating the microreactor. Therefore, our MD simulations focused on the sapphire surface. Figure 5a,b shows the last snapshot of the 0.2 ns MD simulation without and with S adsorption, respectively. Both conditions show that Na_2_WO_4_ becomes droplets on sapphire, with S adsorption resulting in negligible differences. To investigate the distribution of each element in more detail, Figure 5c,d shows histograms along the *x*‐axis for the latter half of the MD simulations corresponding to the conditions shown in Figure 5a,b, respectively. In both cases, the distributions of Na and W atoms exhibit fine peaks and are spread out, suggesting that the atoms of each element diffused on the surface and the distribution was commensurate with the crystal lattice of the sapphire surface. When S atoms were added, the distribution of Na atoms appears to spread in the direction of the S atoms. The time dependence of the diffusion of each element can be inferred from the mean square displacement (MSD) analysis shown in Figure 5e, where panels (i), (ii), (iii), and (iv) show the Na (overall), Na (along the *x* axis), W (overall), and W (along the *x* axis), respectively. The MSD of Na atoms is larger than that of W atoms, indicating that Na atoms diffuse more readily. In addition, the overall MSD of both Na atoms and W atoms does not change appreciably with the addition of S atoms, but the movement of Na atoms in the *x* direction is slightly larger. The local structure also indicates the preferential interaction between Na and S atoms, rather than between W and S atoms. To evaluate the impact of the Al_2_O_3_ crystallinity on the diffusivity of Na, W, and O atoms in the Na─W─O molten phase, we calculated the mean square displacement (MSD) values of each element, as shown in Figure S16 (Supporting Information). No significant differences were observed in the MSD values of each element along the *x* and *y* axes without S atoms. This indicates that the crystallinity of Al_2_O_3_ has no significant effect on the diffusivity of the Na─W─O molten phase. Figure 5f shows the number of S─Na and S─W bonds that are present during the MD simulation with S atom adsorption. While S─W bonds are not formed at all, S─Na bonds are always observed. These results suggest that Na atom diffuses on the sapphire surface, and the supplied S bonds with Na atoms in the early stage of the decomposition of Na_2_WO_4_ crystals. These insights into the behavior of molten Na─W─O─S suggest that the diffusion of the molten droplet can be enhanced by the introduction of S, likely due to effective interactions between Na and S atoms, which may improve the dynamic behavior of the molten droplet.

In the growth regime of the molten droplet (R2), larger single crystals with a higher growth rate can be synthesized. It was essential to form a molten thin film to realize the R2 conditions. The importance of molten thin film formation, which strongly suppresses the nucleation probability, for synthesizing wafer‐scale single crystals of monolayer TMDC with single nuclei has been suggested previously.^[^ ^32^ ^]^ Therefore, developing a strategy for preparing a molten thin film is critical for further improving crystal size.

Regarding the crystal quality of the TMDC crystals grown by the VLS method, the residual source, including alkali, such as Na, which contributes to the strong doping to the TMDC, is an issue for real applications, especially for integrated circuits. Therefore, the next step of the VLS growth TMDCs should be the establishment of cleaning methods to completely remove residues.

### 2.4 Abnormal Ribbon Growth in R3

**Figure 6:**
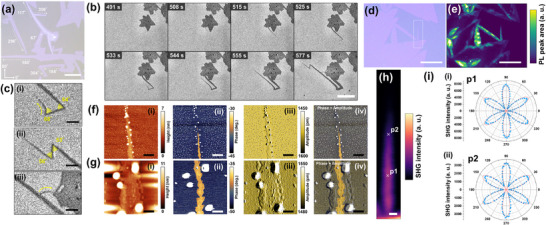
a) Micrographs of WS_2_ ribbons. b) Time‐series micrographs of WS_2_ ribbons. c) Cropped micrographs obtained from in situ observation. d) Micrograph and e) its PL intensity mapping. f,g) AFM images of the i) surface topography, ii) phase, iii) amplitude, and (iii) phase + amplitude with (f) high and (g) low magnification. h) SHG intensity mapping of WS_2_ ribbon corresponding to the area shown in (d) and (f). (i) Polar plot of SHG intensity in p1 and p2 shown in (h). The scale bars shown in (a–h) represent 20, 100, 20, 20, 20, 1 µm, 200 nm, and 1 µm, respectively.

In the R3, we observed an abnormal growth behavior of WS_2_, characterized by a ribbon‐like morphology. A micrograph of a typical ribbon crystal is shown in **Figure** **6**a. Time‐series optical micrographs obtained via in situ observation are shown in Figure 6b, and a movie shown in Movie S5 (Supporting Information), where the emergence of the ribbon‐shaped crystal from an initial 2D domain and its continuous growth are evident. Notably, the growth direction undergoes multiple sudden changes, ultimately resulting in a zigzag‐oriented structure, as shown in (Figure 6c). This morphological evolution can be categorized into two distinct types: abrupt directional changes and gradual curving, resulting in zigzag‐oriented structures and curved ribbons, respectively. In the former case, the change in the growth direction (*θ*_r_) varied ≈60°, ranging from 53° to 68° (Figure 6c). These sudden directional changes are likely attributable to the threefold symmetry inherent to both monolayer WS_2_ and the *c*‐plane sapphire substrate. In contrast, the curvature observed in the ribbons appears to originate from epitaxial growth of WS_2_ on the *c*‐plane sapphire substrate. The WS_2_ ribbons consistently grew along six specific directions (≈0° (or 180°), 60° (or 240°), and 120° (or 300°)) (Figure 6a), which are presumed to correspond to the preferred crystallographic orientations under epitaxial growth conditions. The formation mechanism of the curved ribbon structure can be explained as follows. When the orientation of a WS_2_ crystal immediately after nucleation does not coincide with the thermodynamically most favorable direction, the epitaxial interaction with the *c*‐plane sapphire substrate induces a gradual realignment of the growth axis. This realignment is facilitated by the curvature of the ribbon, which effectively corrected the initial unfavorable orientation. As a result, the WS_2_ ribbons eventually grow along the three most energetically preferred directions. It has been reported that WS_2_ ribbons bend during VLS growth due to the naturally present steps on the SiO_2_/Si substrate.^[^ ^29^ ^]^ Another report on the VLS growth of MoS_2_ nanoribbons similarly mentioned 60° bending at the grain boundaries of the underlying MoS_2_ layer.^[^ ^27^ ^]^ The *c*‐plane sapphire substrate used for WS_2_ growth in our study also exhibits atomic step edges of ≈0.21 nm.^[^ ^57^ ^]^ Thus, it is possible that the WS_2_ ribbons bend at the step edges of the sapphire substrate. We also observed another case of 60° bending, where a WS_2_ ribbon contacted the edge of another WS_2_ domain (Figure S17, Supporting Information). The step height of monolayer WS_2_ was estimated to be ≈0.65 nm (Figure S6, Supporting Information). This observation indicates that atomic‐scale steps, even less than 1 nm, can act as barriers that change the growth direction of WS_2_ ribbons. Therefore, the step edges present on the sapphire substrate could contribute to the 60° bending of WS_2_ ribbons.

The crystal quality of the WS_2_ ribbons was evaluated by PL intensity mapping (Figures 6d,e; Figure S18, Supporting Information). The ribbon exhibits a higher PL intensity than that of the 2D crystal, indicating a better crystal quality of the WS_2_ ribbon than that of the 2D crystal. The surface morphology of the WS_2_ ribbon was analyzed using AFM to acquire height, phase, and amplitude images based on noncontact AFM mode around the region of the tip of the WS_2_ ribbon demarcated in Figure 6d. The height image shows that the residual precursor surrounded the WS_2_ ribbon (Figure 6f(i),g(i)). The phase image clearly reveals the structure of the WS_2_ ribbon (Figure 6f(ii),g(ii)) because the phase image is sensitive to the material properties such as viscoelasticity and the coefficient of friction, enabling different materials to be identified. The amplitude image effectively highlights the surface features and edges (Figure 6f(iii),g(iii)). The composite image of the phase and amplitude images visualizes the coexistent system of the residual precursor and WS_2_ crystal, as shown in Figure 6f(iv),g(iv). The phase image clearly revealed a ribbon structure composed of a chain of small triangular crystals with zigzag‐shaped edges. The minimum width of the constriction points of the ribbon structure was ≈50 nm. Although the growth mechanism of WS_2_ ribbons is not yet fully understood, we propose a possible model in which WS_2_ ribbon nucleation occurs at the tight contact between highly wettable molten droplets and the WS_2_ domain, followed by a chain reaction driven by the accumulation of these molten liquids at the ribbon tip under S‐rich conditions (see Note S3, Supporting Information).

Next, we investigated the crystal orientation of the WS_2_ ribbons using second‐harmonic generation (SHG) spectroscopy of the same region of the tip of the WS_2_ ribbon (Figure 6h,i). The SHG spectra can be obtained from monolayer TMDCs because of the breaking of the inversion symmetry and can be used to determine the crystal orientation and strain of monolayer TMDCs.^[^ ^58^, ^59^, ^60^ ^]^ The SHG intensity map was acquired from the WS_2_ ribbon (Figure 6h). The SHG spectra of the WS_2_ ribbon are shown in Figure S20 (Supporting Information). The flower pattern of the SHG intensity was measured using the parallel polarization technique at positions p1 and p2 shown in Figure 6h (Figure 6i). The flower patterns were fitted by the equation of *I* (θ) = *I*_o_ cos [3(θ − ϕ)]^2^, where θ and ϕ represent the fundamental polarization angle and the rotation angle of the armchair direction relative to the horizontal axis, respectively, and *I*_o_ and ϕ are constants.^[^ ^54^ ^]^ The SHG intensity under a parallel polarization configuration reached its maximum and minimum when the polarization direction aligns with the armchair and zigzag directions of the monolayer WS_2_, respectively. The same orientation of the flower patterns was observed in p1 and p2 (Figure 6i), indicating a continuous crystal orientation of the WS_2_ ribbon within the crystal. The flower patterns indicate that the WS_2_ ribbons grew in an armchair direction.

### 2.5 Molten Particle‐Driven Growth in R4

**Figure 7:**
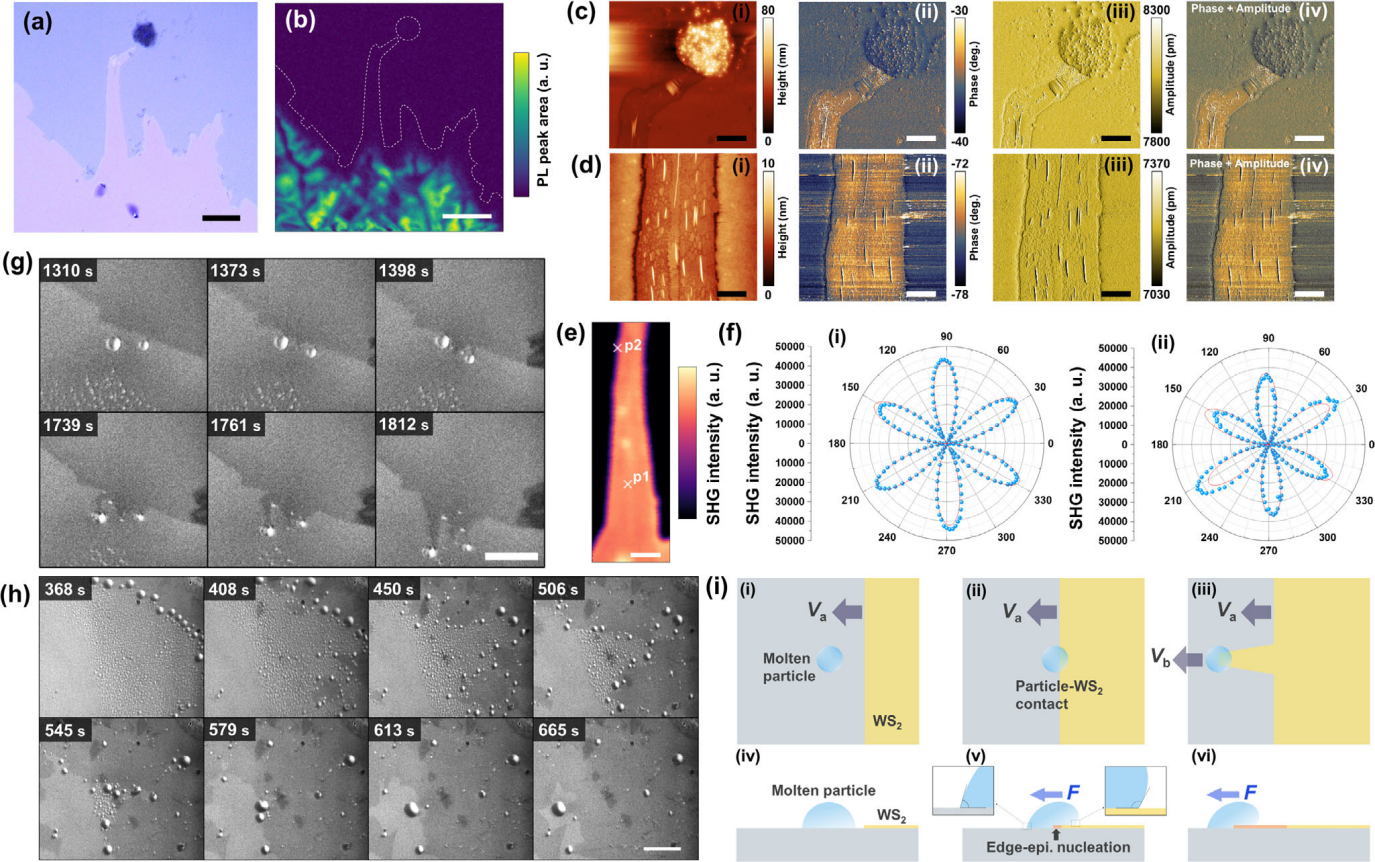
a) Micrograph of molten particle‐driven‐grown WS_2_ ribbon crystal, and its b) PL intensity map. c,d) AFM images of i) surface topography, ii) phase, iii) amplitude and (iii) phase + amplitude around (c) the tip with particles and d) the body of the ribbon. e) SHG intensity map and f) SHG flower pattern at p1 and p2 shown in (e). In situ observation of molten particle‐driven growth of the g) ribbon and h) continuous sheet of WS_2_. (i) Model of the molten particle‐driven growth mechanism. The scale bars shown in (a), (b), (c), (d), (e), (g) and (h) represent 20, 20, 5, 2, 5, 100, and 100 µm, respectively.

In the growth condition with relatively high *C*_W_, leading to a large volume of the Na‐W‐O‐S precursor, we observed the molten particle‐driven growth of a ribbon and a continuous sheet of monolayer WS_2_. Particle‐driven growth has been observed in the VLS regime of TMDC nanoribbons.^[^ ^27^, ^61^, ^62^ ^]^ In this growth regime, as the particles migrated, crystal precipitation occurred along their paths, resulting in the growth of 1D crystal structures. The micrograph of the WS_2_ ribbon grown under these conditions, shown in **Figure** **7**a indicates the presence of large particles around the tip of the WS_2_ ribbon, suggesting the particle‐driven growth of the WS_2_ ribbon. The crystal quality of this ribbon was characterized by PL, AFM, and SHG measurements (Figure 7b–f). The PL map of the WS_2_ ribbon shows that PL was almost absent in the ribbon region, although PL was detected in the sheet region, specifically at the base of the ribbon (Figure 7b; Figure S21, Supporting Information).

The AFM images show the WS_2_ ribbon with particles around the tip of the ribbon (Figure 7c(i)) and the precursor film (Figure 7d(i)). It should be noted that, unlike ribbon growth in R2 (Figure 6f,g), the precursor film was not in close contact with the WS_2_ ribbon edge, and there were gaps between the ribbon and the precursor film (Figure 7d(i)). We observed mottled structures on the WS_2_ ribbon plane, which could correspond to residual precursor particles. Although differences were observed in the phase diagrams of the plane of the ribbon and the outer precursor film, the differences were less pronounced than those observed under R3 conditions (Figure 6f,g(ii)). This could be because the surface of the WS_2_ ribbon was covered by residual precursor. A possible reason for the absence of PL in the WS_2_ ribbon is strong doping from residual precursors,^[^ ^51^ ^]^ which suppresses the PL intensity in addition to the effects of lattice defects. The AFM images showed line‐shaped kink structures aligned parallel to the growth axis in the plane of the WS_2_ ribbons, indicating that lattice strain was induced during the growth process (Figure 7c,d). SHG intensity was detected over the WS_2_ ribbon, indicating a monolayer structure (Figure 7e). The SHG spectra of the WS_2_ ribbons are shown in Figure S20 (Supporting Information). The SHG flower patterns measured at p1 and p2 shown in Figure 7e (Figure 7f) indicate that the WS_2_ ribbon had the same crystal orientation as the armchair axis over the ribbon. The asymmetry of the flower pattern at p2 (Figure 7f(ii)) indicates that the ribbon was partially strained^55^ and could form kink structures, as was observed by AFM (Figure 7c,d).

In situ observation under these growth conditions revealed isolated particles that contributed to the ribbon‐shaped growth (Figure 7g; Movie S6, Supporting Information). The few micrometer‐scale Na─W─O─S molten particles were formed by the introduction of S and initially showed no significant movement (1310–1373 s in Figure 7g). When the edge of the WS_2_ sheet approached and attached to the particles, the particles started to move and grow into WS_2_ ribbons (1398–1812 s). In situ observation revealed that particle‐driven growth was initiated upon the contact between the WS_2_ edge and adjacent particles and proceeded continuously until the particles were entirely consumed. Moreover, we observed continuous film growth by particle‐driven growth for numerous particles (Figure 7h; Movie S7, Supporting Information). Many Na─W─O─S molten particles were formed around the edge of the microreactor by the introduction of the organosulfur (368 s in Figure 7h). Initiated by the nucleation of WS_2_ at a specific site near the edge of the microreactor, contact occurred between the WS_2_ crystal and surrounding particles, triggering particle migration (408 s in Figure 7h). As a result, numerous particles migrated in various directions while precipitating WS_2_ crystals along their paths (450–506 s). Ultimately, a continuous WS_2_ sheet was ultimately formed through subsequent particle fusion and WS_2_ coalescence (545–665 s).

We propose a possible model for the molten particle‐driven growth of WS_2_ ribbons based on a previous report on MoS_2_ nanoribbon growth (Figure 7i).^[^ ^27^ ^]^ Na─W─O─S molten particles remained stationary before contacting the edge of the WS_2_ sheet (Figure 7i(i,iv)). Contact with the edge of the WS_2_ sheet can trigger two phenomena: edge epitaxial nucleation and particle migration (Figure 7i(ii,v), respectively). By inserting the WS_2_ sheet into the particle, asymmetry of the contact angle between the WS_2_ contacting side and the other side was observed. This asymmetric contact angle causes the driving force (*F*) to move toward the lower contact angle side, leading to the migration of particles with ribbon growth (Figure 7i(iii,vi)). Because the particles function as reservoirs of the precursor, they accelerate crystal growth; the ribbon grows at a faster rate (*V*_b_) than the sheet (*V*_a_) (Figure 7i(iii,vi)). A model based on this type of mechanism is highly beneficial for controlling the nanostructure of monolayer TMDCs by leveraging capillary forces at the solid–liquid interface.

## 3 Conclusion

In this study, we achieved the in situ observation of the VLS growth of monolayer WS_2_ crystals within a confined microreactor system. By systematically varying the concentrations of the Na_2_WO_4_ solution and the flow rate of organosulfur, we identified multiple distinct growth regimes, including conventional triangular crystal growth, large hexagonal crystal growth assisted by molten droplets, ribbon‐shaped crystal formation under S‐rich conditions, and molten particle‐driven continuous film growth under high precursor concentrations. In situ observation elucidated the critical role of molten precursor dynamics such as droplet formation, migration driven by surface tension gradients (Marangoni effect), and droplet–crystal interactions during growth. Furthermore, the presence of S lowered the melting point of the Na─W─O molten phase, enhancing the mobility and reactivity of the droplets. These findings provide comprehensive insights into the complex interplay between the precursor supply, confined space effects, and droplet behavior in the VLS growth of TMDCs. This deeper understanding will contribute to the precise control of monolayer TMDC crystal synthesis, enabling the scalable production of high‐quality 2D materials for optoelectronic and quantum device applications.

## 4 Experimental Section

### In Situ Observation of CVD Growth of WS_2_ in the Microreactor

A SiO_2_ (250 nm)/Si substrate and a *c*‐plane sapphire substrate measuring 1 cm^2^ were used as the growth substrates. O_2_ plasma treatment was applied to the SiO_2_/Si substrate to improve its surface wettability. Through spin coating at 3000 rpm, an aqueous solution of Na_2_WO_4_ (1–5 mg mL^−1^) was deposited onto the SiO_2_/Si substrate. The spin‐coated SiO_2_/Si substrate was covered with a *c*‐plane sapphire substrate. The inner spacing at the center of the microreactor (*t*_center_) was estimated to be 1.25, 3.97, and 5.48 µm for *C*_W_ = 1, 3, and 5 mg mL^−1^, respectively, just before the growth process (see Note S4, Supporting Information). An infrared furnace was employed as the heating system, (ADVANCE RIKO), which was equipped with a φ40 quartz tube and window for in situ observation. A zoomable digital microscope with a working distance of 101 mm was used (Asahikogaku).

Table S1 (Supporting Information) shows the growth conditions regarding organosulfur supply and chamber pressure (*P*_chamber_). The quartz tube was evacuated to ≈1.0 Pa using a rotary oil pump. A stainless‐steel tube containing an organosulfur liquid (DTBDS) with an N_2_ bubbling system was connected to the chamber via a needle valve. The flow rate of N_2_ (*F*_N_) and the internal pressure of the stainless‐steel tube (*P*_tube_) were controlled using the needle valve. *F*_S_ was calculated using the equation: *F_S_* = *F_N_* × *P_OS_*/*P_tube_*, where *P*_OS_ is the vapor pressure of DTBDS. *P*_OS_ = 0.1 kPa and listed parameters of *F*_S_, *F*_N_, and *P*_tube_ in the Table S1 (Supporting Information) are used for the caluculation of *F*_S_. The temperature ramp‐up time was set to 10 min. After reaching the desired temperature, the organosulfur‐containing N_2_ gas was introduced. The growth temperature was set to 840 °C, and the average duration of growth was 5–30 min. Immediately following the growth, the temperature was decreased to below 100 °C, and the sample was removed from the chamber. The tone of the captured micrograph images was edited, and a movie was generated using homemade Python code.

### Characterization

The surface morphology was characterized using optical microscopy (OLYMPUS BX53M) and AFM (Park Systems XE7). PL and Raman spectroscopy were performed using a Raman spectrometer (JASCO NRS‐4500 NMDS) equipped with a 532 nm laser. SEM‐EDS (Hitachi High‐Tech SU9000) was employed for elemental analysis. The thermal properties of the molten samples were measured using DTA (SHIMADZU DTG‐60). The SHG spectra were acquired in a parallel configuration in which the fundamental polarization angle was always oriented along the detection angle. A home‐built femtosecond‐pulse laser was used to produce a 250 fs pulse width at a center wavelength of 1560 nm with a 75 mHz repetition rate. The pump beam was focused using a 50X objective lens, and the average pump power on the sample plane was maintained below 10 mW to avoid permanent damage to the TMDC monolayers. A single‐photon avalanche diode and a photon‐counting module were used to measure the second‐harmonic intensity.

### Computational Methods

MD simulations were performed using a universal neural network potential, the preferred potential (PFP),^[^ ^63^ ^]^ which is a pre‐trained machine learning interatomic potential available for various combinations of elements and structures, working on the Matlantis cloud service.^[^ ^64^ ^]^ Universal machine learning force fields such as the PFP enabled the conduction of 4048‐atom simulations involving five elements on a sub‐nanosecond timescale, which is not feasible for density functional theory (DFT) calculations. The PFP model version was “v6.0.0” and the model type “CRYSTAL_U0_PLUS_D3”, which denotes that the training data were calculated using the Vienna ab initio simulation package (VASP)^[^ ^65^, ^66^ ^]^ and Grimme's D3 dispersion correction with the Becke–Johnson damping function^[^ ^67^ ^]^ was added separately to the potential. The DFT calculations for building the PFP were conducted using the Perdew–Burke–Ernzerhof (PBE) functional under the generalized gradient approximation with the projector augmented‐wave (PAW) method without Hubbard U correction. The MD simulations were performed using the ASE package.^[^ ^68^, ^69^ ^]^ The Langevin thermostat was applied for temperature control, and LBFGS algorithm was used to optimize the structure. The initial structure of the MD simulation was built using crystal structures from the Materials Project^[^ ^70^ ^]^ and the visualizing software VESTA.^[^ ^71^ ^]^ The initial structure consisted of a Na_2_WO_4_ crystal placed on the center of an Al_2_O_3_ (0001) surface slab structure. The *x* and *y* axes correspond to the [10‐10] and [11‐20] directions of the Al_2_O_3_ (0001) plane, respectively. The control temperature during the following MD simulations was 1000 K: 1) an 0.1 ns NVT simulation as an equilibration run after the structure optimization from the initial structure; and continuous 0.2 ns NVT simulation as a production run 2) without S and 3) with two S_8_ rings adsorbed onto the structure after the relaxation run (simulation 1) was performed for 0.2 ns after a structure optimization. The initial structure and the last structure of each MD simulation (1–3) are provided as Supporting Information text files, along with video files of the MD simulations (1–3) in mp4 format.

## Conflict of Interest

The authors declare no conflict of interest.

## Author Contributions

H.S. and Y.S. contributed equally to this work. H.S. and Y.H. designed the experiments and supervised the project. Y.S., S.A., and H.S. performed the TMDC growth. Y.S., S.F., Y.T., and H.S. performed the characterization. K.H. performed the MD simulations. Y.S., K.H., and H.S. analyzed the data. All the authors discussed the results and commented on the manuscript.

## Supporting information



Supporting Information

Supplemental Movie 1

Supplemental Movie 2

Supplemental Movie 3

Supplemental Movie 4

Supplemental Movie 5

Supplemental Movie 6

Supplemental Movie 7

Supplemental Movie 8

Supplemental Movie 9

Supplemental Movie 10

## Data Availability

The data that support the findings of this study are available from the corresponding author upon reasonable request.
