# Supplementary material for: Inside the Microreactor: In Situ Real‐Time Observation of Vapor–Liquid–Solid Growth of Monolayer TMDCs
Source: Adv Sci (Weinh). 2025 Dec 12;13(11):e16784. doi: 10.1002/advs.202516784 (PMC12931242; doi:10.1002/advs.202516784)
Supplement: Supplementary file 1 — Supporting Information [file ADVS-13-e16784-s011.pdf]

Inside the Microreactor: In Situ Real-Time Observation of Vapor–Liquid–Solid Growth of Monolayer TMDCs

Hiroo Suzuki[*1,2,3†], *Yutaro Senda*[1†], *Kaoru Hisama*[4‡], *Shuhei Aso*[3], *Yuta Takahashi*[5], *Shun Fujii*[5], *Yasuhiko Hayashi*[1,2,3]

[1]Graduate School of Environment, Life, and Natural Science and Technology, Okayama University, Okayama 700-8530, Japan

[2]Faculty of Environment, Life, and Natural Science and Technology, Okayama University, Okayama 700-8530, Japan

[3]Department of Electrical and Communication Engineering, School of Engineering, Okayama University, Okayama 700-8530, Japan

[4]Research Initiative for Supra-Materials, Shinshu University, Nagano 380-8553, Japan

[5]Department of Physics, Faculty of Science and Technology, Keio University, Yokohama 223-8522, Japan

[†]These authors contributed equally

[‡]Current affiliation: Preferred Networks, Inc., 1-6-1, Otemachi, Chiyoda-ku, Tokyo 100-0004, Japan

*Email: hiroo.suzuki@okayama-u.ac.jp

Supplementary Note S1: Growth side of WS_2 crystals

We considered the reason why the WS_2 crystals preferentially grow on the top sapphire side. The temperature difference between the top and bottom substrates could play a crucial role in this face-selective growth. We assume that the temperature of the top sapphire substrate is lower than that of the bottom SiO_2/Si substrate because sapphire has a lower thermal conductivity (~30 W m^{-1} K^{-1}) compared to SiO_2/Si (~140 W m^{-1} K^{-1}), and also a lower infrared absorbance.

To experimentally verify this hypothesis, we performed WS_2 growth using a microreactor formed by placing two SiO_2/Si substrates on the top and bottom, with a different heating system equipped with an infrared light source positioned above the sample stage (Figure S2a). This system allows us to establish a temperature gradient from top to bottom, where the temperature of the top substrate becomes higher than that of the bottom one. Using this setup, we found that the WS_2 crystals preferentially grew on the bottom side (Figure S2b,c), which is opposite to the result obtained with the in situ observation system equipped with infrared heating sources on both the upper and lower, providing more uniform heating of the top and bottom substrates (Figure S3). These results indicate that the preferred growth face of WS_2 is determined by the temperature difference between the top and bottom substrates, even in the absence of differences in surface materials. The Na_2WO_4 sources are likely transferred from the bottom SiO_2/Si side to the top sapphire side, facilitated by the temperature gradient. This occurs through the evaporation of Na_2WO_4 from the bottom substrate and its subsequent condensation on the upper sapphire substrate.

To confirm this phenomenon, we conducted an experiment as shown in Figure S4. A microreactor was configured by covering a Na_2WO_4–spin-coated SiO_2/Si substrate with a sapphire substrate, and the system was heated to 840 °C without supplying the organosulfur precursor. As a result, particles of Na_2WO_4 were observed on the top sapphire substrate, indicating that Na_2WO_4 can be transferred from the bottom to the top side. This phenomenon likely accounts for the WS_2 growth observed on the top sapphire substrate.

Supplementary Note S2: In situ observation without microreactor

We conducted in situ observations of WS_2 growth on a SiO_2/Si substrate without using a microreactor (Figure S8 and Supplementary Movie S8). To suppress the evaporation of Na_2WO_4, which significantly disturbs WS_2 growth by depleting the W source, we lowered the growth temperature from the standard 840 °C to 700 °C. It should be noted that WS_2

crystals cannot grow at 840 °C without the microreactor, indicating that the microreactor plays a critical role in suppressing Na_2WO_4 evaporation during growth. Upon introducing the organosulfur precursor, large droplets formed in the high-Na_2WO_4-volume region near the substrate edge, as shown in Figure S8a (t_g = 0–6 s). Continuous reactions with the organosulfur led to the formation of multilayer WS_2 crystals from these large droplets, as shown in Figure S8a (t_g = 10–50 s) and Figure S8b. Unlike the growth with a microreactor, these droplets did not migrate inward across the substrate. In the low-Na_2WO_4-volume region at the substrate center, the thin Na_2WO_4 precursor films transformed into small WS_2 crystals (Figure S8c). The distinct difference between the growths with and without a microreactor is the presence or absence of molten droplet transport toward the inner region. This difference likely arises from the spatial distribution of the organosulfur supply: it is supplied isotropically to the molten droplets without the microreactor, whereas it is supplied anisotropically within the microreactor. The resulting sulfur concentration gradient from the edge to the center may enhance anisotropic droplet motion in the microreactor environment.

Supplementary Note S3: Growth mechanism of zigzag-shaped WS_2 ribbons

Under S-rich conditions, highly wettable molten droplets with low contact angles were observed, as shown in Figure S13a,b. These droplets were in tight contact with the edge of the WS_2 domain (Figure S13a), and similar droplets were also observed at the edges of WS_2 ribbons (Figure 6f,g). Furthermore, molten droplets were found at the tips of WS_2 ribbons, as shown in Figure 6f. From these observations, we infer that the tight contact of highly wettable molten droplets with the WS_2 domain could promote ribbon-like growth. Figure S19 illustrates a possible model for the ribbon-like growth. Abnormal local nucleation may occur from tightly contacted molten droplets with high S concentration at the WS_2 domain (Figure S19a(i)). As the nuclei appear, the molten droplets could be pushed out and accumulate at the tip edge (Figure S19a(ii)), which in turn induces additional nucleation at the tip. By repeating this process, the WS_2 ribbon could grow continuously through a chain reaction (Figure S19a(iii)). The formation of the ribbon-like structure with a zigzag-shaped edge can be attributed to the preferential appearance of S-zigzag edges under S-rich conditions (Figure S19b) [S1,2].

[S1] S. Wang, Y. Rong, Y. Fan, M. Pacios, H. Bhaskaran, K. He, J. H. Warner, *Chem. Mater.* **2014**, *26*, 6371.
[S2] D. Cao, T. Shen, P. Liang, X. Chen, H. Shu, *J. Phys. Chem. C* **2015**, *119*, 4294.

Supplementary Note S4: Estimation of gap distance in microreactor

To roughly estimate the gap distance between the top and bottom substrates before the process, we measured the interference fringes of the microreactor, as shown in Figure S22a,b. The inner spacing at the center (x_{center}) of the microreactor (t_{center}) can be estimated using the following relations:

$$\Delta t = \lambda/2 \quad (1)$$
$$\alpha = \Delta t/\Delta x \quad (2)$$
$$t_{\text{center}} = \alpha \times x_{\text{center}} \quad (3)$$

where λ, α, and Δx represent the wavelength, the contact angle between the top and bottom substrates, and one period of the interference fringes, respectively. Assuming λ = 550 nm, we obtained t_{center} = 1.25, 3.97, and 5.48 µm for C_W = 1, 3, and 5 mg/mL, respectively (Figure S22c).

The value of t_{center} tends to increase with increasing C_W, indicating that the nonuniformity of the Na_2WO_4 thickness increases with higher precursor concentration. These values are much larger than the Na_2WO_4 thickness measured by AFM (Figure S1). This mismatch between the estimated gap distance and the measured Na_2WO_4 thickness may be attributed to the nonuniform film thickness caused by edge accumulation inherent to the spin-coating process. However, since the estimated gap distances correspond to the state before annealing, the actual gap is expected to become narrower during heating due to melting of Na_2WO_4, likely approaching the Na_2WO_4 thickness measured by AFM. Estimating the actual gap distance during the growth process remains an important future task for understanding the detailed growth behavior within the microreactor.

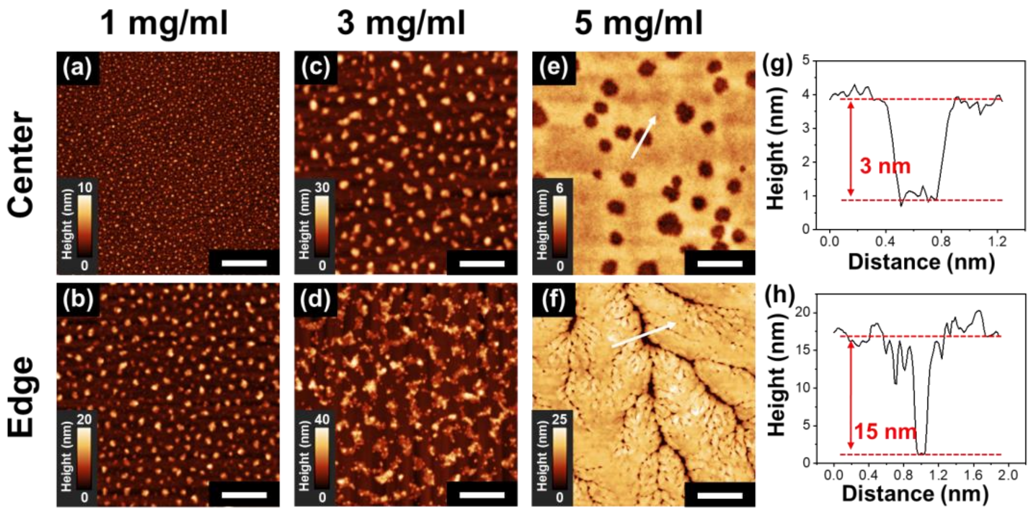

Figure S1. (a–f) AFM topography images of Na_2WO_4 films spin-coated on SiO_2/Si substrates with different coating solution concentrations (C_W): (a,b) 1 mg/mL, (c,d) 3 mg/mL, and (e,f) 5 mg/mL, taken at the (a,c,e) center and (b,d,f) edge regions. (g,h) Height profiles along the white arrows shown in (e) and (f). Scale bars in (a–f) represent 1 µm.

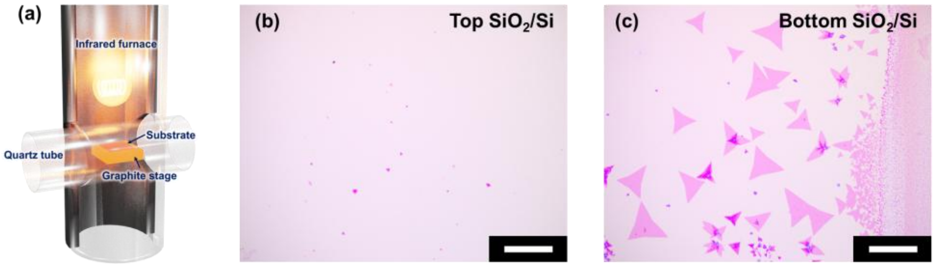

Figure S2. (a) Schematic of infrared furnace equipped infrared light source on the upper side of the sample stage. (b, c) Micrographs of WS_2 on the (b) top and (c) bottom SiO_2/Si substrates, grown using a microreactor composed of two SiO_2/Si substrates. The scale bars in (b,c) are 100 µm.

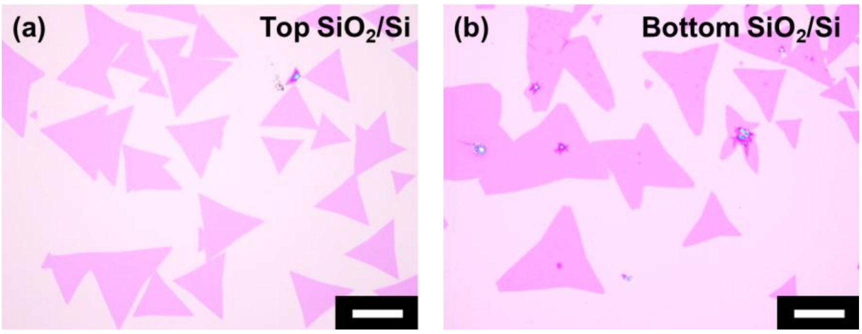

Figure S3. (a,b) Micrographs of WS_2 on the (a) top and (b) bottom SiO_2/Si substrates, grown using a microreactor composed of two SiO_2/Si substrates with in situ observation system. The scale bars in (a) and (b) are 100 µm and 200 µm, respectively.

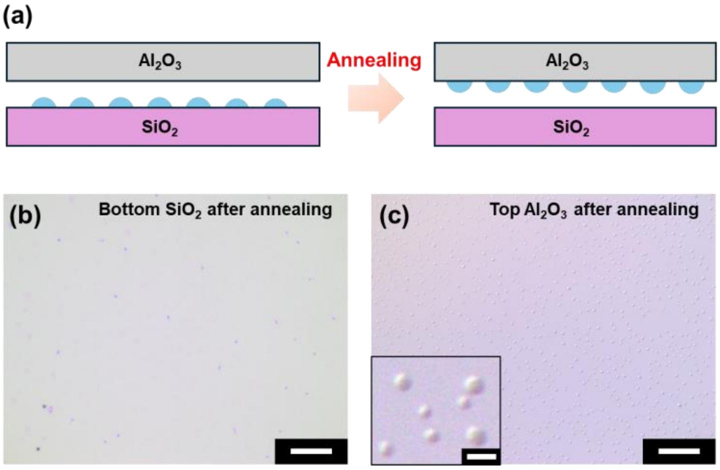

Figure S4. (a) Schematic of transfer of Na_2WO_4 sources from bottom SiO_2 to sapphire side. (b,c) Micrographs of bottom SiO_2/Si and top sapphire substrate after annealing of microreactor. The scale bars shown in (b), (c), and inset in (c) represent 20, 20, and 2 µm, respectively.

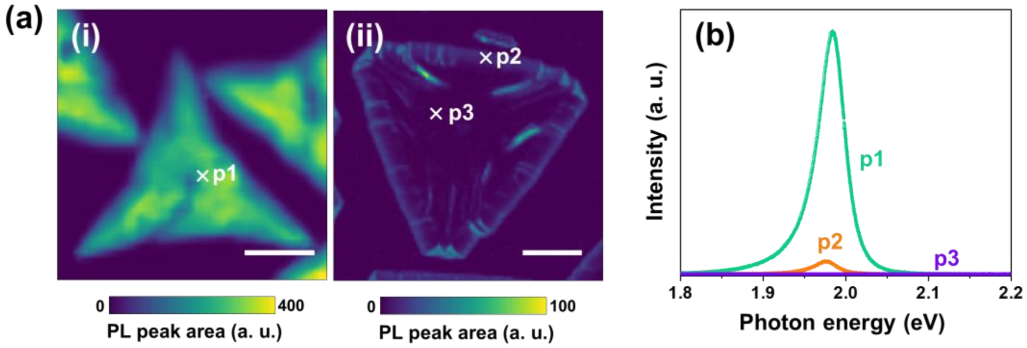

Figure S5. (a) PL maps of WS_2 crystals grown via (i) R1 and (ii) R2. The PL spectra corresponding to p1, p2, and p3 are shown in (a). The scale bars in (a) are (i) 10 μm and (ii) 20 μm.

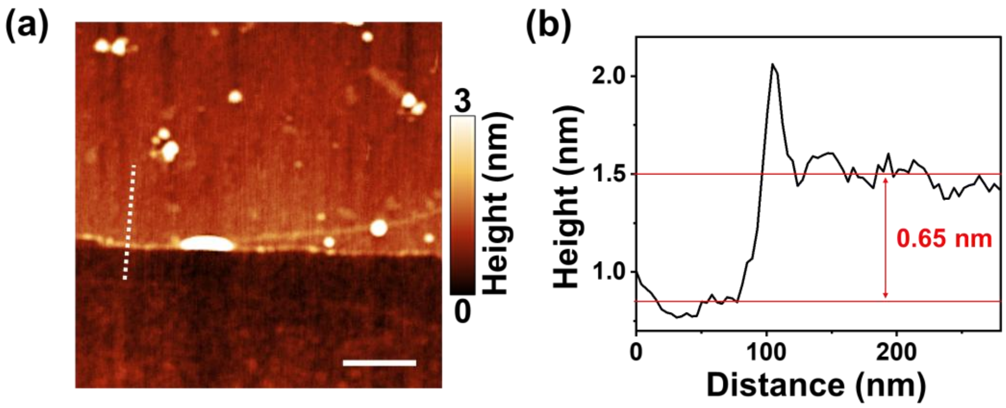

Figure S6. (a) Height image of monolayer WS_2 grown via R1 and (b) its height profile along white dot line shown in (a). Scale bar shown in (a) is 200 nm.

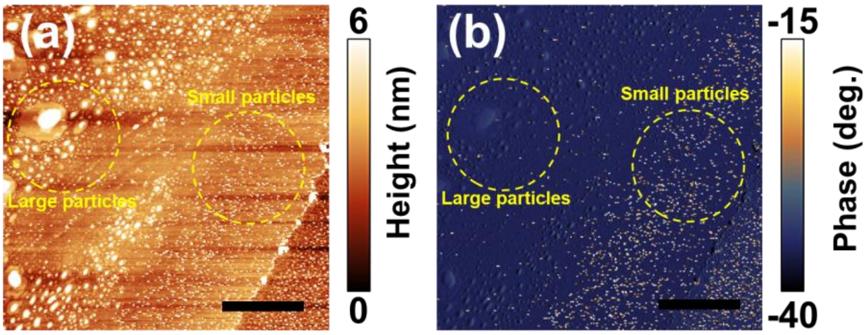

Figure S7. (a) AFM topography images of Na-W-O-S particles after WS_2 growth process under sulfur-rich condition. (b) Height profile of along white arrows shown in (a). Scale bars shown represent 500 nm.

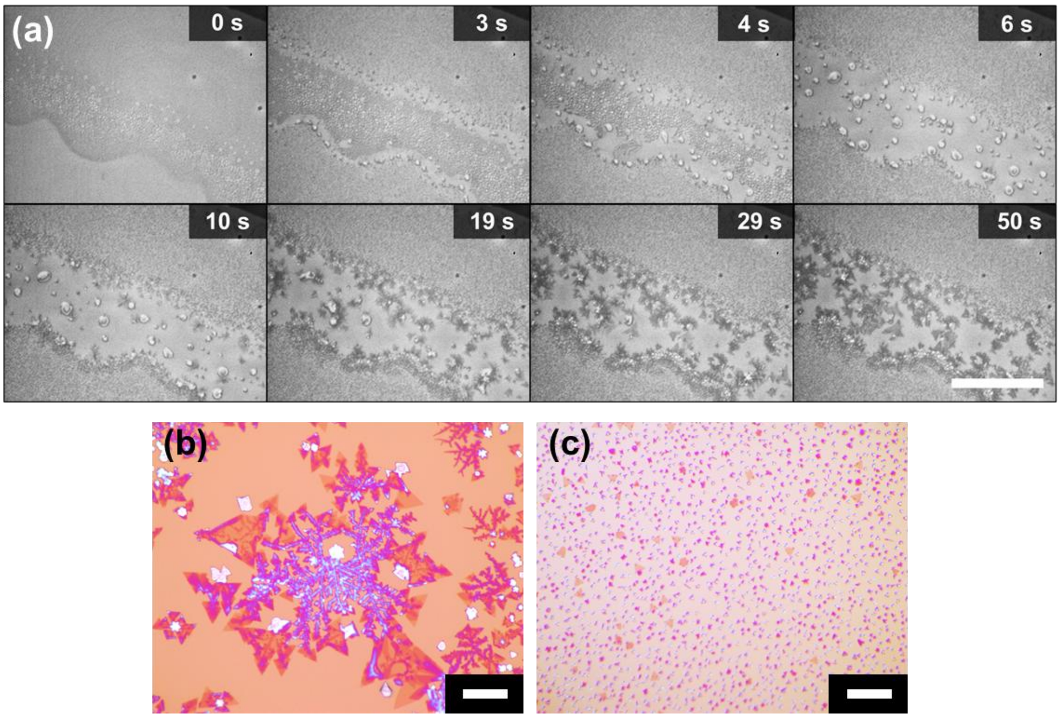

Figure S8. (a) Time-series micrographs of WS_2 growth without microreactor. (b,c) Micrographs of WS_2 crystals grown at (b) high-Na_2WO_4-volume and (c) low-Na_2WO_4-volume area. The scale bars shown in (a), (b), and (c) represent 200, 20, and 20 μm, respectively.

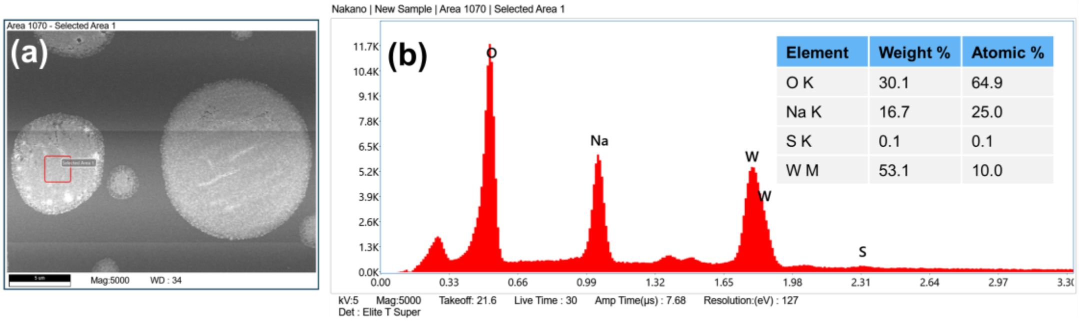

Figure S9. (a) SEM image of precursor particles and (b) its EDS spectra taken from the red enclosed area shown in (a). The inset in (b) presents a table of elemental percentages. The scale bar in (a) is 5 µm.

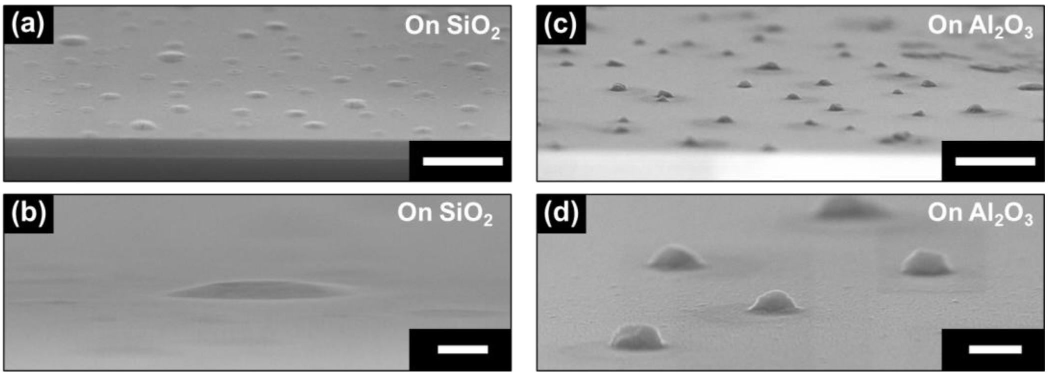

Figure S10. (a–d) Cross-sectional SEM images of Na_2WO_4 particles after vacuum annealing on (a,b) SiO_2/Si and (c,d) sapphire substrates, taken at (a,c) low and (b,d) high magnifications. The scale bars shown in (a), (b), (c) and (d) represent 1, 0.1, 1, and 0.2 µm, respectively.

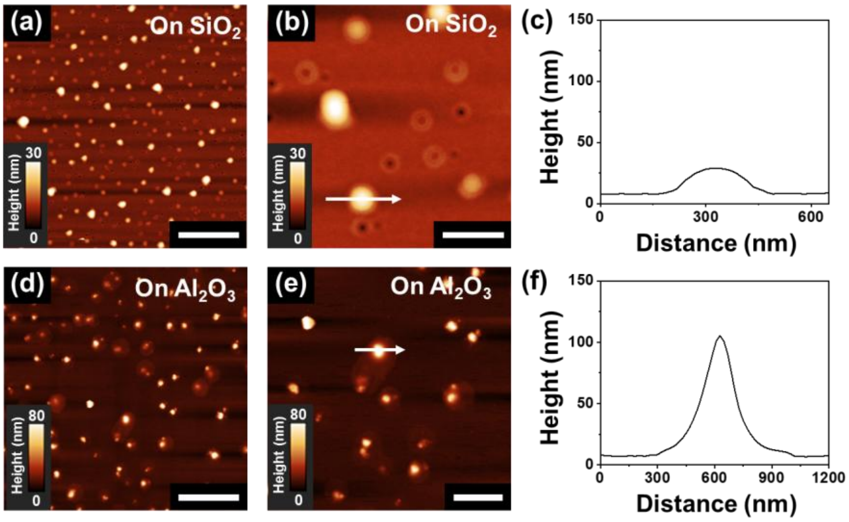

Figure S11. (a-e) AFM topography images of Na_2WO_4 particles after vacuum annealing on (a,b) SiO_2/Si and (d,e) sapphire substrates, taken at (a,d) low and (b,e) high magnifications. (c,f) Height profile of along white arrows shown in (b) and (e), respectively. Scale bars shown in (a), (b), (d), and (e) represent 2.5, 0.5, 2.5, and 1 μm, respectively.

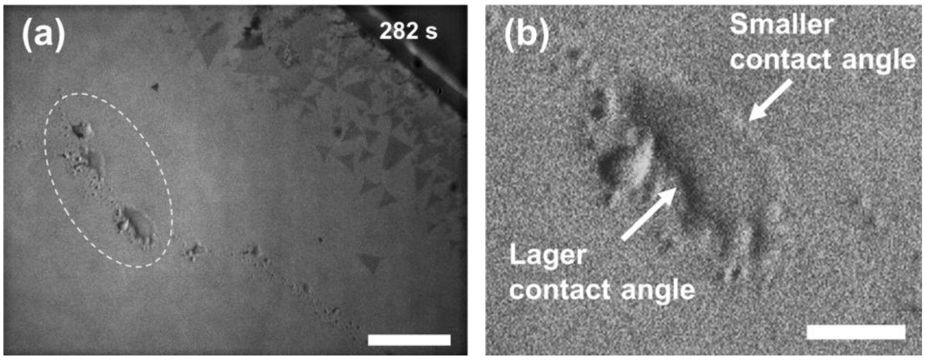

Figure S12. Micrograph of droplets with uneven contact angles, enclosed by the white dashed circle, taken during in situ observation. The scale bars in (a,b) represent 200 and 50 μm, respectively.

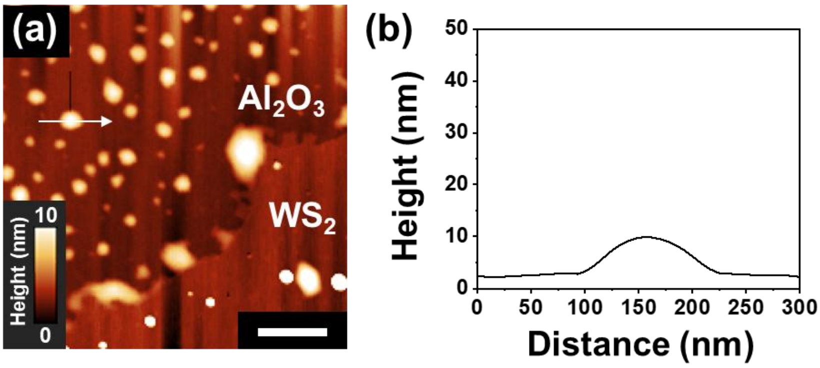

Figure S13. (a) AFM topography images of Na-W-O-S particles after WS_2 growth process under sulfur-rich condition. (b) Height profile of along white arrows shown in (a). Scale bar shown in (a) represents 500 nm.

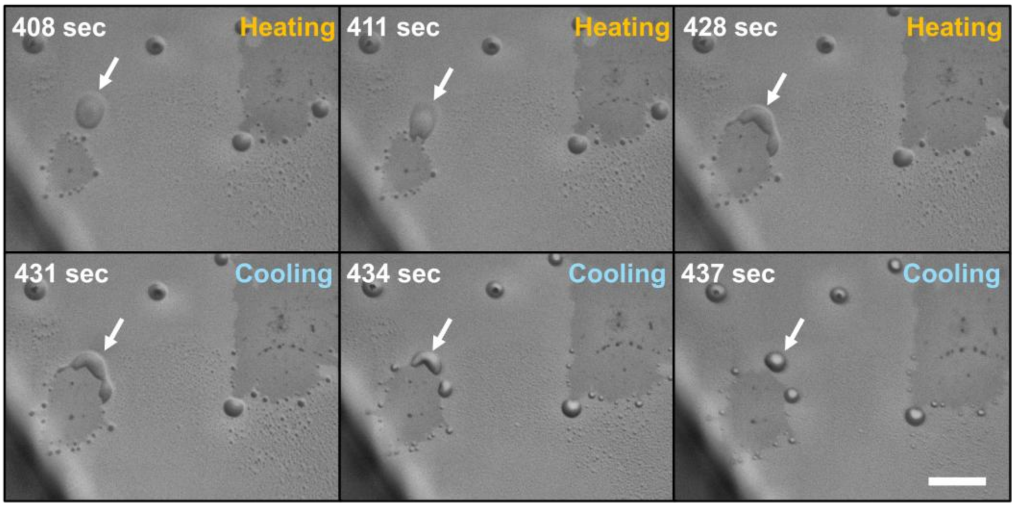

Figure S14. Time-series micrographs showing migration and morphological change of molten droplet before and after cooling, taken by in-situ observation. Scale bar shown is 100 um.

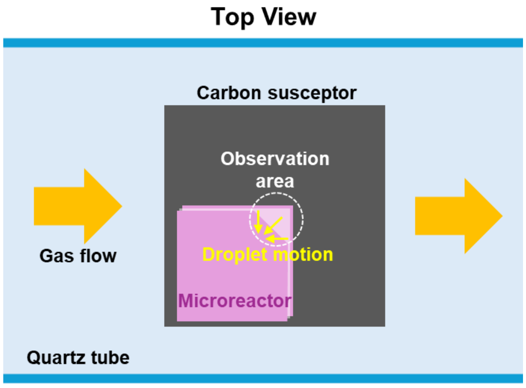

Figure S15. Schematic layout of in-situ observation from top view.

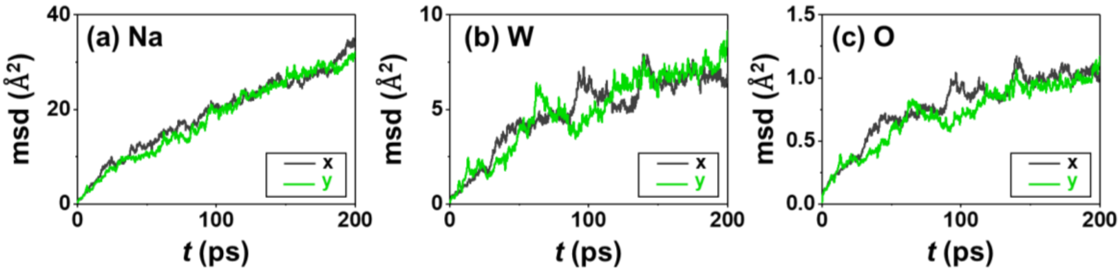

Figure S16. (a-c) MSD as a function of time during the MD simulation of (a) Na, (b) W, and (c) O atoms along the x and y axis.

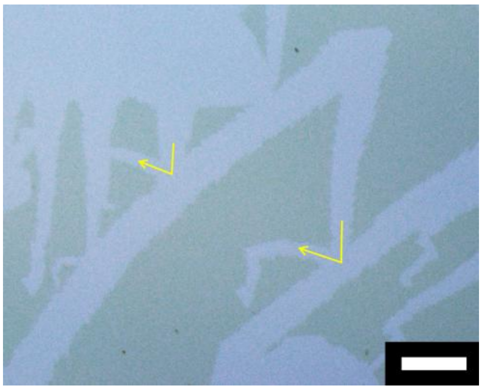

Figure S17. Micrograph of WS_2 ribbons with 60° bending at contact with another WS_2 domains. The scale bar represents 20 µm.

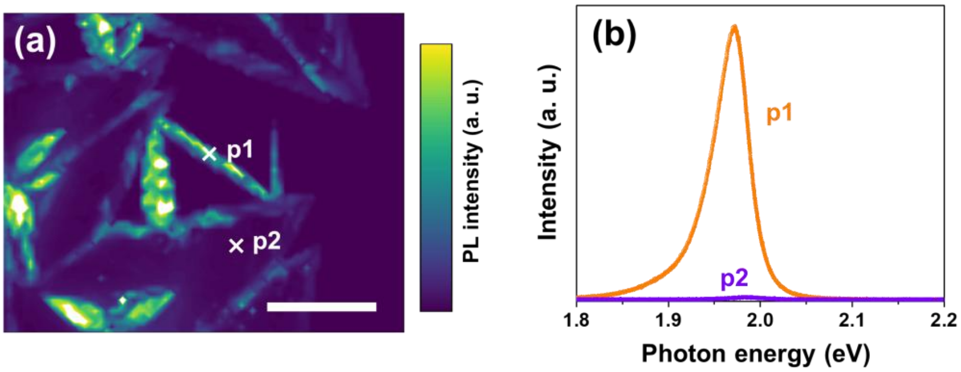

Figure S18. (a) PL maps of WS_2 crystals grown via R3. The PL spectra corresponding to p1 and p2 are shown in (a). The scale bar in (a) is 20 μm.

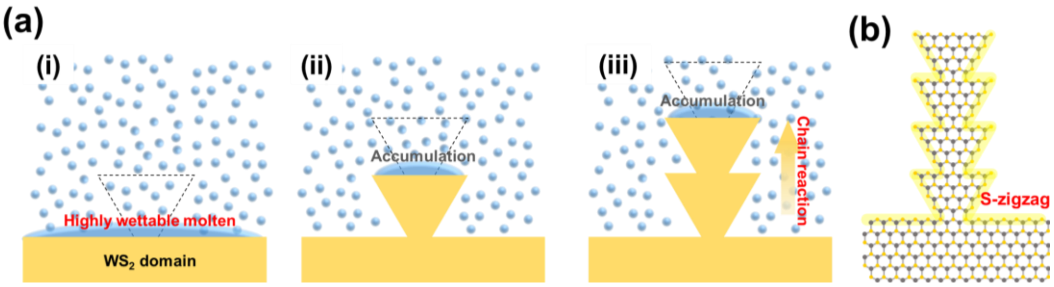

Figure S19. (a) Schematic of model for ribbon-shape growth. (b) Schematic of crystal structure of WS_2 ribbon with S-zigzag edges.

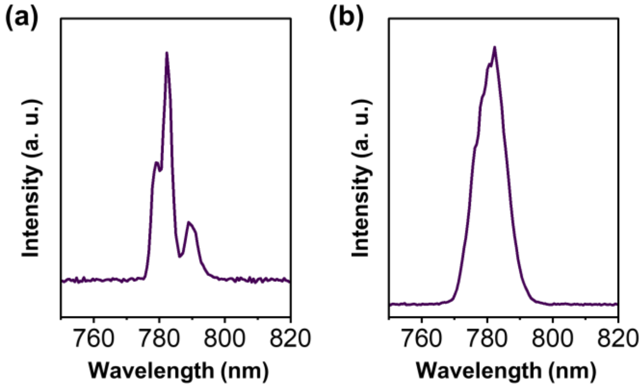

Figure S20. SHG spectra of WS_2 grown via (a) R3 and (b) R4.

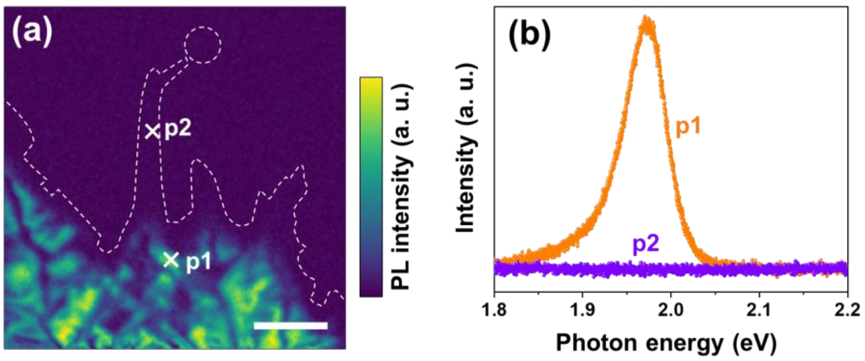

Figure S21. (a) PL maps of WS_2 crystals grown via R4. The PL spectra corresponding to p1 and p2 are shown in (a). The scale bar in (a) is 20 μm.

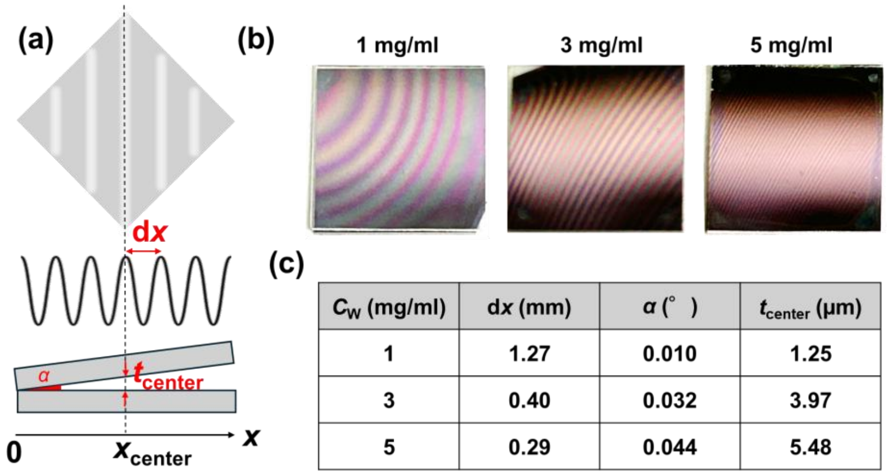

C_W (mg/ml)	dx (mm)	α (°)	t_{center} (μm)
1	1.27	0.010	1.25
3	0.40	0.032	3.97
5	0.29	0.044	5.48

Figure S22. (a) Model of interference fringes of the microreactor. (b) Photographs of microreactors with different C_W and (c) the table of their staking properties.

Table S1. Growth conditions of F_S, F_N, P_{tube}, and $P_{chamber}$.

F_S (sccm)	F_N (sccm)	P_{tube} (kPa)	$P_{chamber}$ (Pa)
0.13	25	19	27
0.18	50	28	45
0.24	100	41	73
